# Influence of Reynolds Number on Multi-Objective Aerodynamic Design of a Wind Turbine Blade

**DOI:** 10.1371/journal.pone.0141848

**Published:** 2015-11-03

**Authors:** Mingwei Ge, Le Fang, De Tian

**Affiliations:** 1 State Key Laboratory of Alternate Electrical Power System with Renewable Energy Sources, North China Electric Power University, Beijing, P. R. China; 2 Sino-French Engineering School, Beihang University, Beijing, P.R. China; Tsinghua University, CHINA

## Abstract

At present, the radius of wind turbine rotors ranges from several meters to one hundred meters, or even more, which extends Reynolds number of the airfoil profile from the order of 10^5^ to 10^7^. Taking the blade for 3MW wind turbines as an example, the influence of Reynolds number on the aerodynamic design of a wind turbine blade is studied. To make the study more general, two kinds of multi-objective optimization are involved: one is based on the maximum power coefficient (*C*_*Popt*_) and the ultimate load, and the other is based on the ultimate load and the annual energy production (*AEP*). It is found that under the same configuration, the optimal design has a larger *C*_*Popt*_ or *AEP* (*C*_*Popt*_//*AEP*) for the same ultimate load, or a smaller load for the same *C*_*Popt*_//*AEP* at higher Reynolds number. At a certain tip-speed ratio or ultimate load, the blade operating at higher Reynolds number should have a larger chord length and twist angle for the maximum *C*_*popt*_//*AEP*. If a wind turbine blade is designed by using an airfoil database with a mismatched Reynolds number from the actual one, both the load and *C*_*popt*_//*AEP* will be incorrectly estimated to some extent. In some cases, the assessment error attributed to Reynolds number is quite significant, which may bring unexpected risks to the earnings and safety of a wind power project.

## Introduction

**Fig 1:**
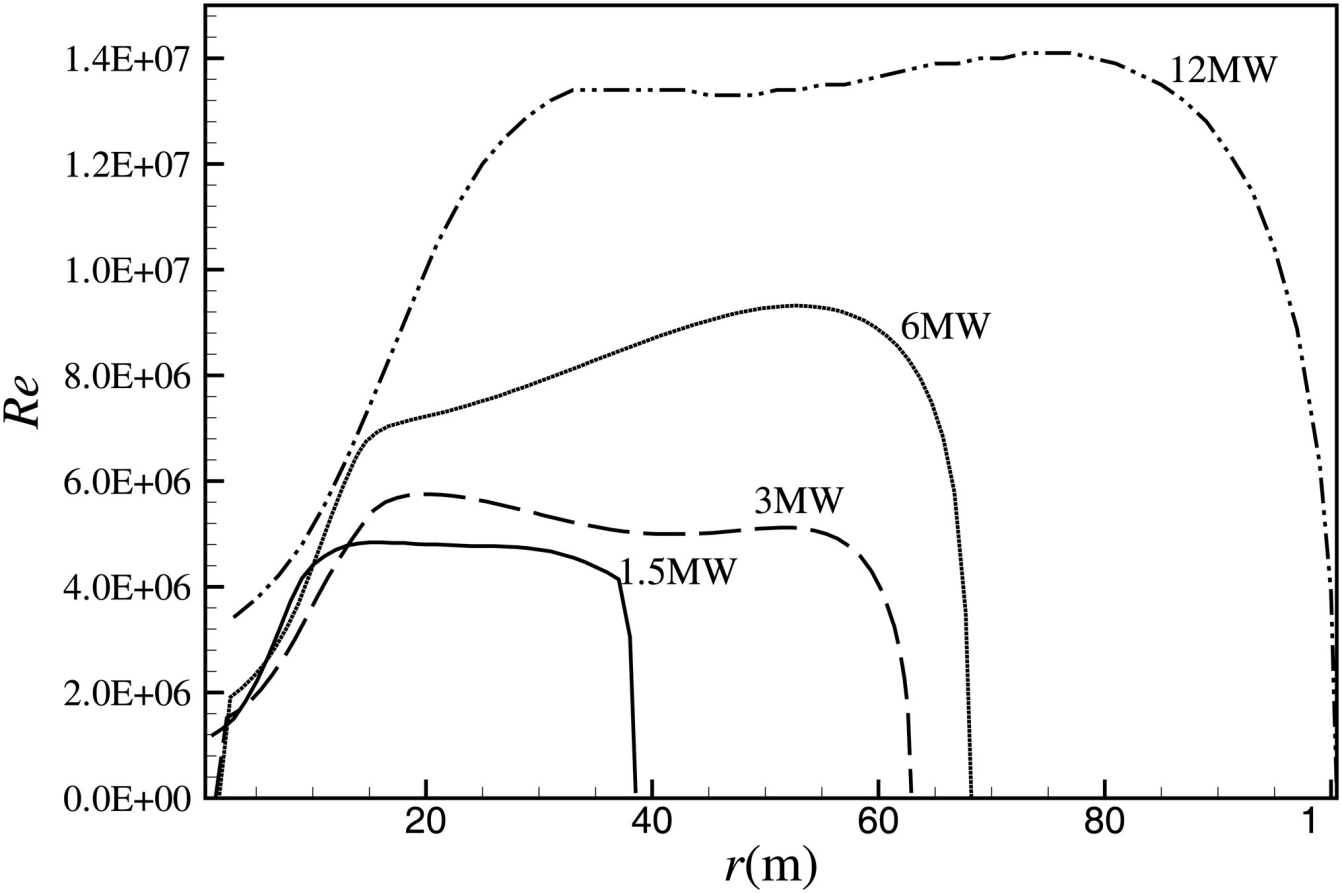
Distribution of Reynolds number along the blade length for four typical MW-class wind turbines [7].

Currently, the business operations of wind power companies are mainly based on onshore MW-class wind turbines, such as 1.5MW, 2MW, and 3MW wind turbines. But driven by economic efficiency, there is a great demand for very large offshore wind turbines [1]. Recently, many types of 5MW-8MW wind turbines have been successfully designed and put into commercial operation around the world, such as the Repower 5MW wind turbine, Siemens 6-MW wind turbine, and the Vestas 8-MW wind turbine (V164). Many larger wind turbines are also at the preliminary design stage [2–4], such as the 10MW-class wind turbine supported by the National High Technology Research and Development Program of China, and the 20MW wind turbine being developed in Energy Research Centre of the Netherlands [5–6]. It can be clearly seen that large-scale wind turbines have become the development trend of wind power. At present, the radius of wind turbine rotors ranges from several meters to one hundred meters, or even more, which extends Reynolds number of the airfoil profile from the order of 10^5^ to 10^7^. Here, Reynolds number of the airfoil profile is defined as Re = *Uc*/*ν*, where, *U* is the relative velocity of airfoil profile, *c* is the chord length, and *ν* is the kinematic viscosity. Fig 1 shows the distribution of Reynolds number along blades for different MW-class wind turbines at the rated condition [7]. Taking the 12MW wind turbine that is in the preliminary design in United Power Company as an example, the blade is 100 meters long, corresponding to Reynolds number of around 1.3×10^7^.

As a dimensionless number expressing the ratio of inertial forces to viscous forces, Reynolds number has a great influence on the flow characteristics. Hence, airfoils at different Reynolds numbers exhibit distinctive performance, directly affecting the aerodynamic design of wind turbine rotors. As Reynolds number changes, the blade shape needs to be adjusted to ensure that the blade operates under optimal conditions, thus bringing a new topic to the design of large-scale wind turbines. However, research on the effect of Reynolds number is still very limited. It is a pity that due to the high cost and limitation of wind tunnel, for large wind turbines, there is little test data available for the wind turbine airfoils at Reynolds number higher than 4×10^6^ [8]. The predictive values of airfoil analysis codes, such as XFOIL/RFOIL [9–10] and Navier-Stokes solvers, provide designers with an effective way for establishing airfoil database at high Reynolds number. By using the numerical code XFOIL, Bak [11] has studied the aerodynamic performance of several airfoil families at different Reynolds numbers from 2×10^5^ to 1.2×10^7^. It is reported that the maximum *c*_*l*_/*c*_*d*_ increases rapidly until around *Re* = 2×10^6^, but increases at a slower rate beyond *Re* = 2×10^6^. By the software RFOIL, Ceyhan [12] and Ge et al. [7] have numerically investigated the performance of several airfoils at high Reynolds number, as well as the influence of Reynolds numbers on the optimal shape of a wind turbine blade, aiming to keep the power coefficient as high as possible. But generally, many aspects including the aerodynamic efficiency, ultimate load, weight, cost, noise, etc., need to be considered in the aerodynamic design of a wind turbine rotor [13–18].

As an extension of the study by Ge et al. [7] which only focuses on the optimal power coefficient, multi-objective optimization of a 60m blade for 3MW wind turbines is performed in the present study at different Reynolds numbers, and in particular, the influence of Reynolds number on the optimal shape, ultimate load and *C*_*Popt*_//*AEP* are analyzed. For simplification, stress is only laid on the two key issues, namely the aerodynamic performance and the ultimate load that are closely correlated with the output power and mechanical cost [13–14]. To make the study more general, two methods of optimization are considered: one is based on the ultimate *M*_*xy-r*_ and *C*_*Popt*_, and the other is based on the ultimate *M*_*xy-r*_ and *AEP*. The Blade Element Momentum (BEM) Theory [19–20], which is widely used for design of wind turbine rotors in scientific research and industry, is adopted in this work. Although the three-dimensional flow characteristics, including the rotational effect, the separation of vortices and the span-wise flow, are ignored due to the two-dimensional assumptions, reliable and accurate results are obtained from BEM theory by some sophisticated modifications [21–23].

The main contents of this paper are as follows: in Section 2, the settings and procedure for multi-objective optimization of the 60-m blade for 3MW wind turbines are outlined; in Section 3 and Section 4, the influence of Reynolds number on multi-objective optimization of the blade are analyzed based on the ultimate *M*_*xy-r*_ and *C*_*Popt*_ as well as the ultimate *M*_*xy-r*_ and *AEP*, respectively; in Section 5, design uncertainty at mismatched Reynolds number is discussed; and finally, in Section 6, the research findings and conclusions are presented.

## Settings and Procedure for Multi-Objective Optimization of the Blade

Airfoil selection is very important for the design of a wind turbine blade, and there are many applicable excellent airfoils, such as the airfoils of NREL (S), Risø, FFA, DU, and NACA6 [24–28]. Six types of airfoils with the thickness ranging from 40% to 18%, including DU00-W2-401, DU00-W2-350, DU97-W-300, DU91-W2-250, NACA 63421 and NACA 64618, are adopted in the present study, following many industry blades [1, 2, 5].

### 2.1 Main parameters of the blade

**Fig 2:**
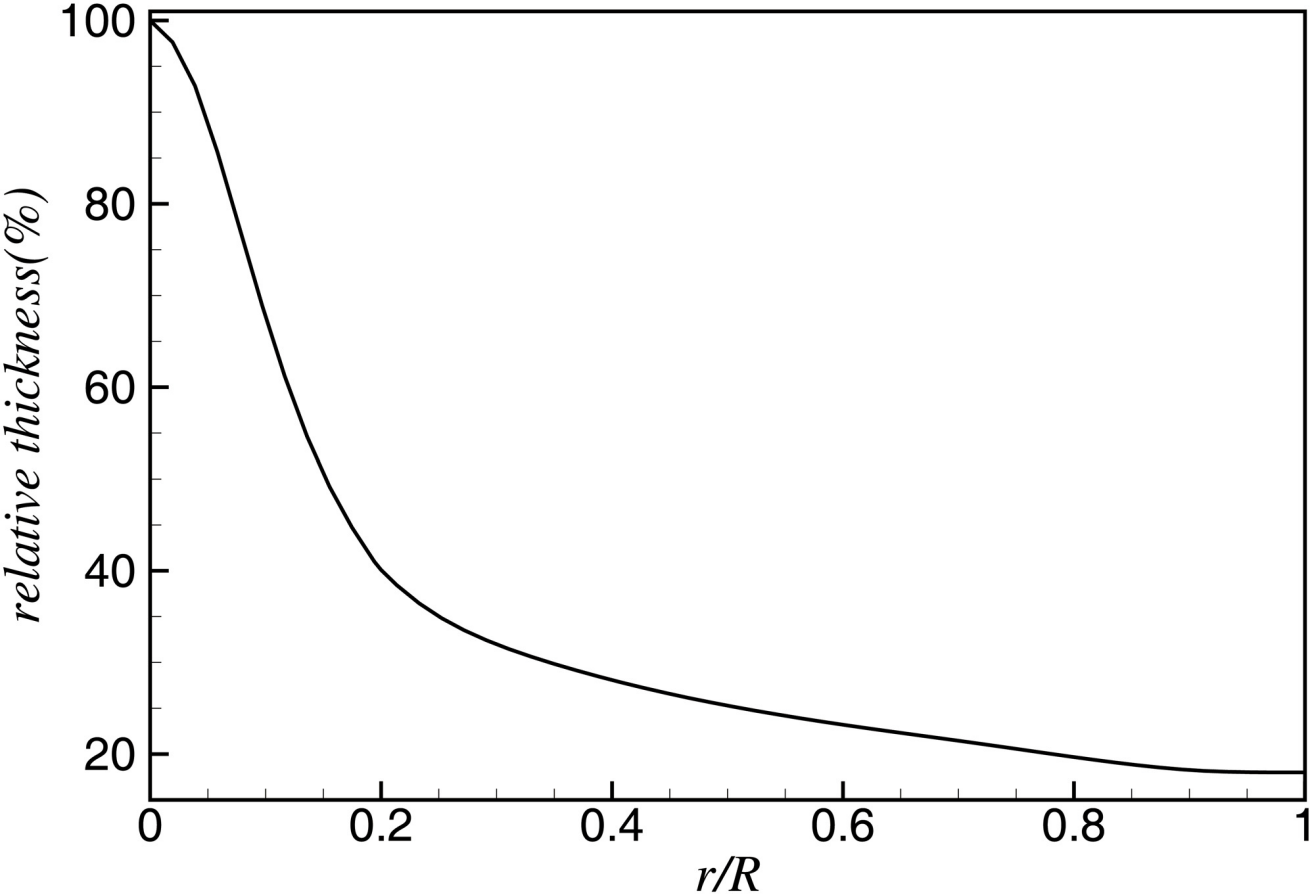
Relative thickness distribution of airfoil profiles along the 60-m blade.

Fig 2 shows the relative thickness distribution of the blade airfoil. From the root to location of the maximum chord length, the relative thickness of airfoil ranges from 100% to 40%, where the main focus is the structure safety and reliability. From location of the maximum chord length to the tip, where most of the power and load are produced, airfoils with a relative thickness of 40% to 18% are used. In blade optimization, distribution of the relative thickness is kept unchanged, only to reveal the influence of Reynolds number. The chord distribution is optimized from the maximum chord location to the tip, as the twist angle is optimized from the root to tip. Design parameters for the blade in this study are shown in Table 1.

**Table 1:** Design parameters for the blade.

Radius of the blade root: 2.2m	Rated power: 3MW
Radius of the hub: 1.4m	Cut in wind speed: 3m/s
Air density: 1.225kg/m^3^	Cut out wind speed: 25m/s
Viscous coefficient: 1.82E-5	The maximum chord length: 4.0m
Cone angle: -3 degree	Location of the maximum chord: 12m
Tilt angle: 5 degree	The minimum rotational speed of rotor: 7.35rpm
Length of blade: 60m	The rated rotational speed of rotor: 12.6rpm

### 2.2 Airfoil database at different Reynolds numbers

**Fig 3:**
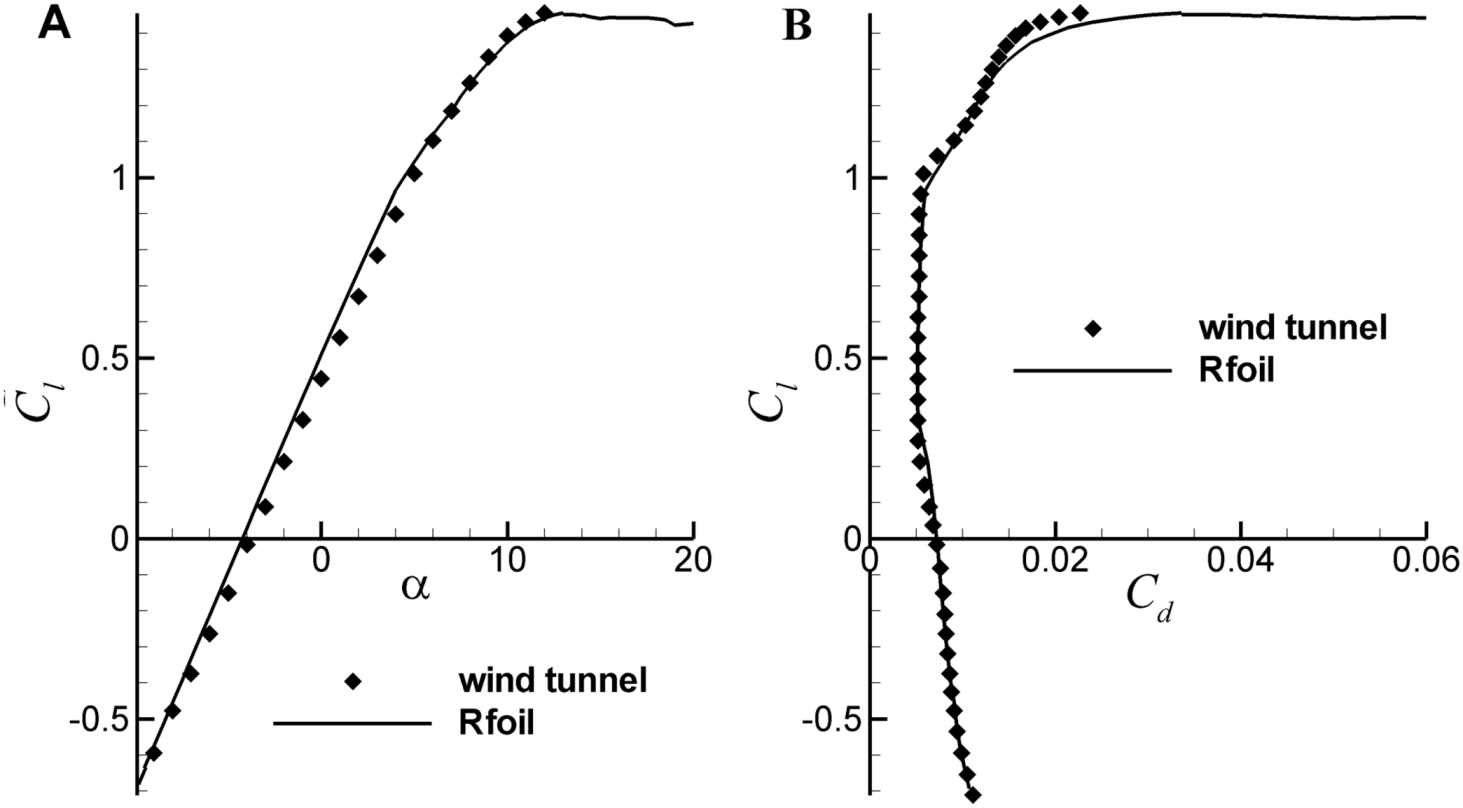
Comparison between the prediction results and the measurements. (A) *C*_*l*_ of NACA64618, (B) *C*_*d*_ of NACA64618 (RFOIL data is multiplied by a factor of 1.09)

Here, it is mainly concerned with Reynolds number from 10^6^ to 10^7^, which covers most of the commercial wind turbine blades. Fig 3A shows a comparison between the RFOIL predicted lift coefficients of airfoil NACA64618 at Re = 3×10^6^ and the measurements from Langley low-turbulence pressure tunnel [29]. As an excellent numerical code, RFOIL can well predict the main part of the lift coefficient. For the drag coefficient, an additional drag of 9% is suggested by Timmer [29] to correct the RFOIL data; with the addition of this factor, the drag coefficient from RFOIL also shows a good agreement with the test data, as shown in Fig 3B. Hence, in the following study, the lift coefficient of airfoils is directly calculated from RFOIL, while the drag coefficient is obtained by an artificial adjustment of the RFOIL predicted data.

**Fig 4:**
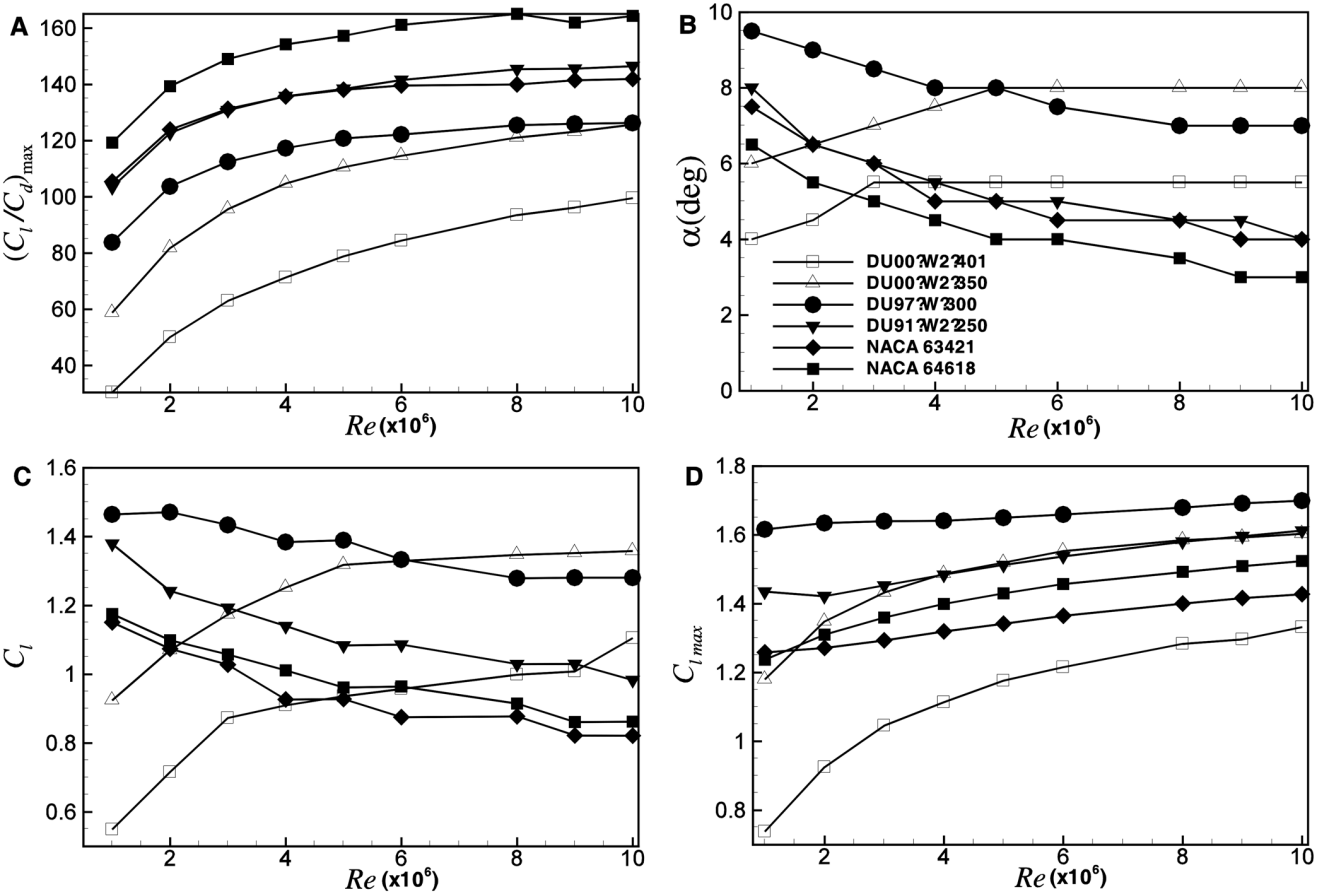
Influence of Reynolds number on performance of the six airfoils (DU00-W2-401, DU00-W2-350, DU97-W-300, DU91-W2-250, NACA 63421 and NACA 64618). (A) the maximum *C*_*l*_/*C*_*d*_ vs. *Re*, (B) the corresponding angle of attack at the point of maximum *C*_*l*_/*C*_*d*_ vs. *Re*, (C) the corresponding *C*_*l*_ at the point of maximum *C*_*l*_/*C*_*d*_ vs. *Re*, (D) the maximum *C*_*l*_ vs. *Re*

*C*_*l*_ and *C*_*d*_ for the six airfoils at Reynolds number between Re = 1×10^6^ and Re = 1×10^7^ are evaluated by RFOIL. In RFOIL, the effect of rotation on airfoil characteristics is taken into consideration, for a better maximum lift coefficient and post-stall prediction [30]. For other regular airfoils, in the region of small angles, *C*_*l*_ increases with Reynolds number, while *C*_*d*_ decreases with Reynolds number, thus inducing a larger *C*_*l*_ */C*_*d*_ at higher Reynolds number, as shown in Fig 4A. As Bak [10] stated, the airfoil performance is most sensitive around Re = 2×10^6^, and the Reynolds number effect becomes smaller with the increase of Reynolds number. Fig 4B and 4C show *α* and *C*_*l*_ at the point of maximum *C*_*l*_/*C*_*d*_, respectively. Interestingly, the thicker airfoils behave differently from the thinner ones. For thicker airfoils, the maximum *C*_*l*_/*C*_*d*_ increases with Reynolds number, but the corresponding angle of attack *α* and lift coefficient *C*_*l*_ decrease. In the region of larger angle of attack, the stall angle increases with Reynolds number, which means the maximum *C*_*l*_ increases at higher Reynolds number, as shown in Fig 4D. Since the Reynolds number effect is quite similar for airfoils at Reynolds number between 1×10^6^ and 1×10^7^, it is mainly concerned with the airfoil database at Re = 3×10^6^ and Re = 6×10^6^ in this paper. A comparison is to be made in detail between optimization using the airfoil database at Re = 3×10^6^ and at Re = 6×10^6^.

In assessment of the blade performance and load via BEM, the lift and drag coefficients at angles of attack from -180° to 180° should be provided. For angles of attack larger than the stall angle, the empirical model proposed by Viterna and Corrigan [31], which is to modify the aerodynamic parameters of *C*_*l*_ and *C*_*d*_ in the stall regime, is used in the present study, following Vaz et al. [32].

For angles of attack larger than the stall angle,

$$C_{l}=\frac{C_{d,\text{max}}}{2}\text{sin}2\alpha +K_{l}\frac{{\text{cos}}^{2}\alpha }{\text{sin}\alpha }$$ (1)

$$C_{d}=C_{d,\text{max}}{\text{sin}}^{2}\alpha +K_{d}\text{cos}\alpha$$ (2)

Where,

$$K_{l}=(C_{l,s}-C_{d,\text{max}}\text{sin}\alpha_{s}\text{cos}\alpha_{s})\frac{\text{sin}\alpha_{s}}{{\text{cos}}^{2}\alpha_{s}}$$ (3)

$$K_{d}=\frac{C_{d,s}-C_{d,\text{max}}{\text{sin}}^{2}\alpha_{s}}{\text{cos}\alpha_{s}}$$ (4)

Here, *C*_*d*,max_ is the maximum drag coefficient in the stall region.

For the aspect ratio *Ar*≤50,

$$C_{d,\text{max}}=1.11+0.018\mu_{0}$$ (5)

For *Ar*≥50, *C*_*d*,max_ = 2.01;

Where, the aspect ratio *Ar* is defined as:

$$\mu_{0}=\frac{R-r_{hub}}{c(r)}$$ (6)

### 2.3 Procedure of the multi-objective optimization

As design variables, distributions of the chord and twist angle are both parameterized by Bezier curves. Seven and six control points are respectively used in parameterization of the chord and twist angle distributions.

$$\begin{array}{l} r_{c\text{max}}=x_{c1}<x_{c2}<x_{c3}<x_{c4}<x_{c5}<x_{c6}=x_{c7}=r_{tip}\\ c_{\text{max}}=y_{c1}=y_{c2}<y_{c3}<y_{c4}<y_{c5}<y_{c6}<y_{c7}=c_{tip}\\ r_{t\text{max}}=x_{t1}<x_{t2}<x_{t3}<x_{t4}<x_{t5}<x_{t6}=r_{tip}\\ \beta_{\text{max}}=y_{t1}=y_{t2}<y_{t3}<y_{t4}<y_{t5}<y_{t6} \end{array}$$ (7)

Where, *x*_*c*_, *y*_*c*_, *x*_*t*_, *y*_*t*_ are the horizontal and vertical coordinate values of the control points for the chord length and twist angle, respectively. To meet such practical requirements as the manufacturing procedure, transport limitations and structural design, some artificial constraints are applied empirically on the control points, where *r*_*cmax*_ = 12, *r*_*tip*_ = 60, *c*_*max*_ = 4.0, *c*_*tip*_ = 0.02, *r*_*tmax*_ = 7, *β*_max_ = 15. The coordinate values of the control points are taken as sixteen optimization variables:

$$X=(x_{c2},\ldots ,x_{c5},y_{c3},\ldots ,y_{c6},x_{t2},\ldots ,x_{t5},y_{t3},\ldots ,y_{t6})$$ (8)

It is mainly concerned with two objectives related with loads and power efficiency in this study. Generally, the flap-wise and edge-wise moments of blade are the reference loads for determination of the rigidity and strength in blade structural design. Hence, the ultimate moment of the blade root *M*_*xy-r*_ is set to be one of the optimization objectives:

$$Y_{1}=\text{min}(M_{xy-r})$$ (9)

The power coefficient *C*_*P*_, or the annual energy production (*AEP*) is usually maximized for better power efficiency of a wind turbine. In some cases, to optimize *C*_*P*_ for blades is a simple way to optimize *AEP* [10]. But a good *AEP* doesn’t need to reach a very high *C*_*P*_ at a single point on the *C*_*P*_-*λ* curve. Hence, two different kinds of optimization are adopted in the present study, to reveal the Reynolds number effect on the aerodynamic design of a wind turbine blade. In the first kind, *C*_*P*_ is set to be one of the two optimization objectives, while in the second kind, *AEP* is set to be one of the optimization objectives:

$$Y_{2,1}=\text{max}(C_{P})$$ (10)

$$Y_{2,2}=\text{max}(AEP)$$ (11)

The advanced BEM theory proposed by Lanzafame (2007) is used for assessment [21]. For axial induction factors greater than 0.4, the BEM theory cannot yield reliable results. Therefore, correlation is necessary to eliminate the unfaithful results. When *a*>0.4, and *F*<1, the correlation proposed by Buhl [33] is adopted:

$$a=\frac{18F-20-3\sqrt{C_{x}(50-F)+12F(3F-4)}}{36F-50}$$ (12)

Base on BEM theory, the tangential induction factor can be easily obtained:

$$b=\frac{1}{2}\left(\sqrt{1+\frac{4a(1-a)}{\lambda^{2}\mu^{2}}-1}\right)$$ (13)

With the induction factors *a* and *b*, the aerodynamic load on the blade element (the lift *L* and the drag *D*) can be calculated, as well as *M*_*xy-r*_, *P*, *C*_*Mxy-r*_ and *C*_*P*_:

$$\begin{array}{l} M_{x-r}=\int \frac{1}{2}\rho cU_{\infty }^{2}C_{y}r\text{d}r,\;M_{y-r}=\int \frac{1}{2}B\rho cU_{\infty }^{2}C_{x}r\text{d}r\\ P=NM_{x-r}\Omega ,\;M_{xy-r}=\sqrt{M_{x-r}^{2}+M_{y-r}^{2}} \end{array}$$ (14)

$$C_{Mxy-r}=M_{xy-r}/\rho AU_{\infty }^{2}R,\;C_{P}=2P/\rho AU_{\infty }^{3}$$ (15)

**Fig 5:**
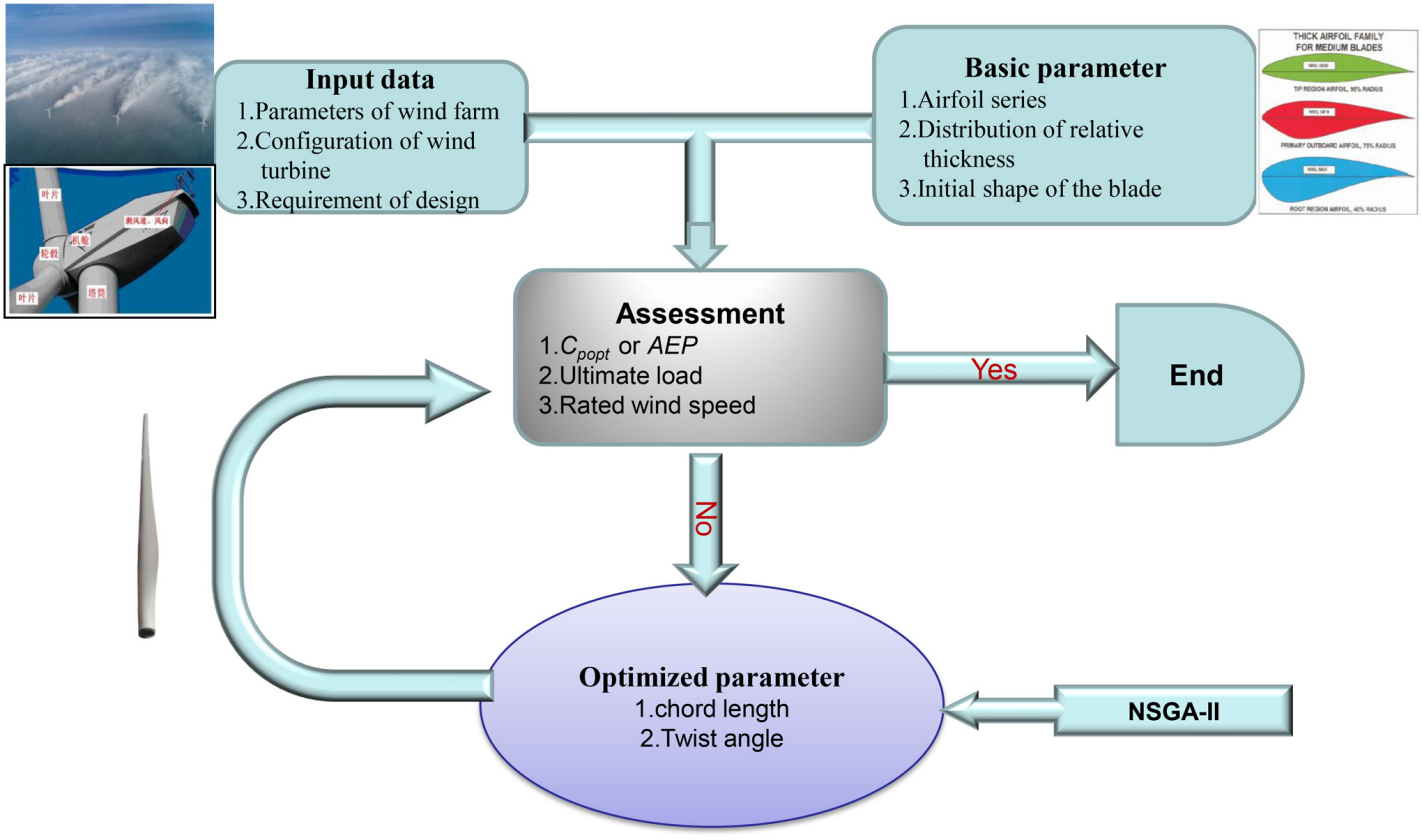
Procedure for aerodynamic design of a wind turbine blade.

*AEP* is predicted by using a Weibull distribution with a mean wind speed of 7.5m/s. The hub height is 120m, and the constant C = 2.0. Fig 5 shows the procedure for aerodynamic design of a wind turbine blade. The multi-objective Generic algorithm NSGA-II [34] is introduced for optimization.

## Optimization of the Wind Turbine Blade Based on *C*_*Popt*_ and *M*_*xy-r*_ at *Re* = 3×10^6^ and *Re* = 6×10^6^

**Fig 6:**
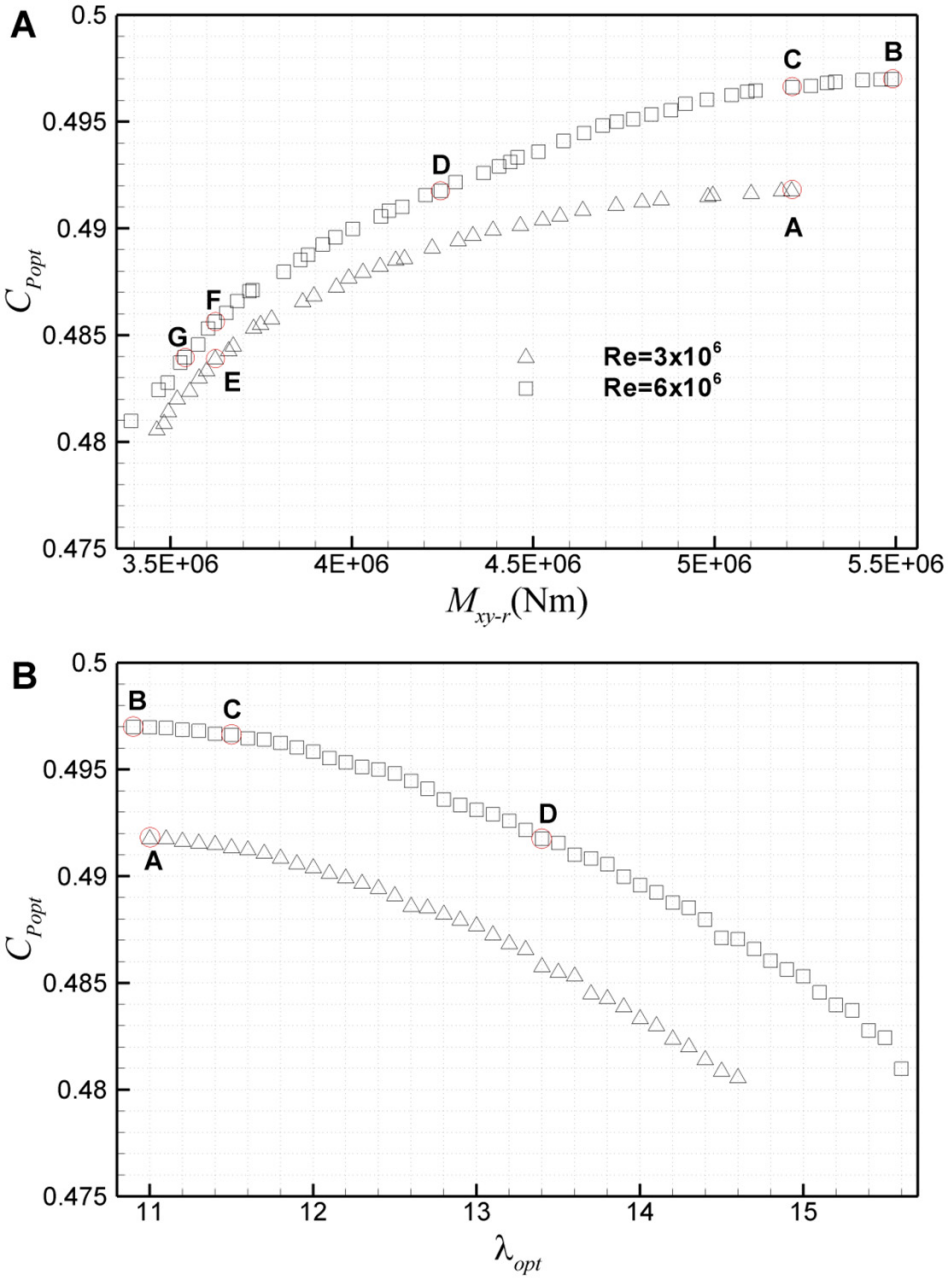
Pareto frontiers based on the ultimate *M*_*xy-r*_ and *C*_*Popt*_ in planes of (A) *C*_*popt*_ *-M*_*xy-r*_ and (B) *C*_*popt*_-*λ*_*opt*_.

Fig 6 shows the Pareto frontiers of optimization based on the ultimate *M*_*xy-r*_ and *C*_*Popt*_ with NSGA-II method. As shown in Fig 6A, the Pareto frontier using airfoil database at Re = 3×10^6^ is generally on the lower-right side of that at Re = 6×10^6^, which means that design based on the airfoil database at higher Reynolds number has a greater power coefficient *C*_*popt*_ at the same ultimate load *M*_*xy-r*_, and a smaller load at the same *C*_*popt*_. However, it should be noted that there is an extra Pareto frontier in the large-load region for higher Reynolds number, such as the points on the right side of C. To reveal the Reynolds number effect, four points A, B, C and D on Pareto frontiers are studied in detail. Points A and B correspond to the largest *C*_*Popt*_ on the two frontiers at Re = 3×10^6^ and Re = 6×10^6^, respectively; C represents the design on Pareto frontier at Re = 6×10^6^ with the same ultimate *M*_*xy-r*_ as A, and D represents that with the same *C*_*Popt*_. To show the optimal tip-speed ratios (*λ*_*opt*_ *)* of these designs, the Pareto frontier is also given in the *C*_*Popt*_-*λ*_*opt*_ plane, as shown in Fig 6A. Interestingly, *λ*_*opt*_ is almost the same for points A and B. It can be clearly seen that both *C*_*Popt*_ and the ultimate *M*_*xy-r*_ decrease with *λ*_*opt*_ on both Pareto frontiers.

### 3.1 Comparison of A and B

For an ideal blade without any constraint but only using an airfoil profile, all the blade elements tend to operate at the largest *C*_*l*_/*C*_*d*_ point, to reduce the losses induced by drag, as well as achieving the best *C*_*P*_ at a single point [20]. Hence, the chord length and twist angle can be approximately calculated from Eqs (16) and (17) by maximization of the power coefficient, since the drag losses only contribute a little to the optimal shape. In the two equations, *C*_*l*_ and *α* are the lift coefficient and angle of attack corresponding to the largest *C*_*l*_/*C*_*d*_ for a given airfoil at a certain Reynolds number. As is shown, in the ideal blade design, the chord length is inversely proportional to the operating *C*_*l*_ at a certain *λ*, since both the induction factors *a* (*a* = 1/3), and *b* (*b* = *a* (1-*a*)/*λ* ^2^ *μ* ^2^) are constants under the optimal conditions.

$$\frac{Nc}{2\pi R}\lambda C_{l}=\frac{4\lambda^{2}\mu^{2}b}{\sqrt{{\left(1-a\right)}^{2}+\lambda^{2}\mu^{2}{\left(1+b\right)}^{2}}}$$ (16)

$$\beta =\text{arctan}\left(\frac{1-a}{\lambda \mu (1+b)}\right)-\alpha$$ (17)

**Fig 7:**
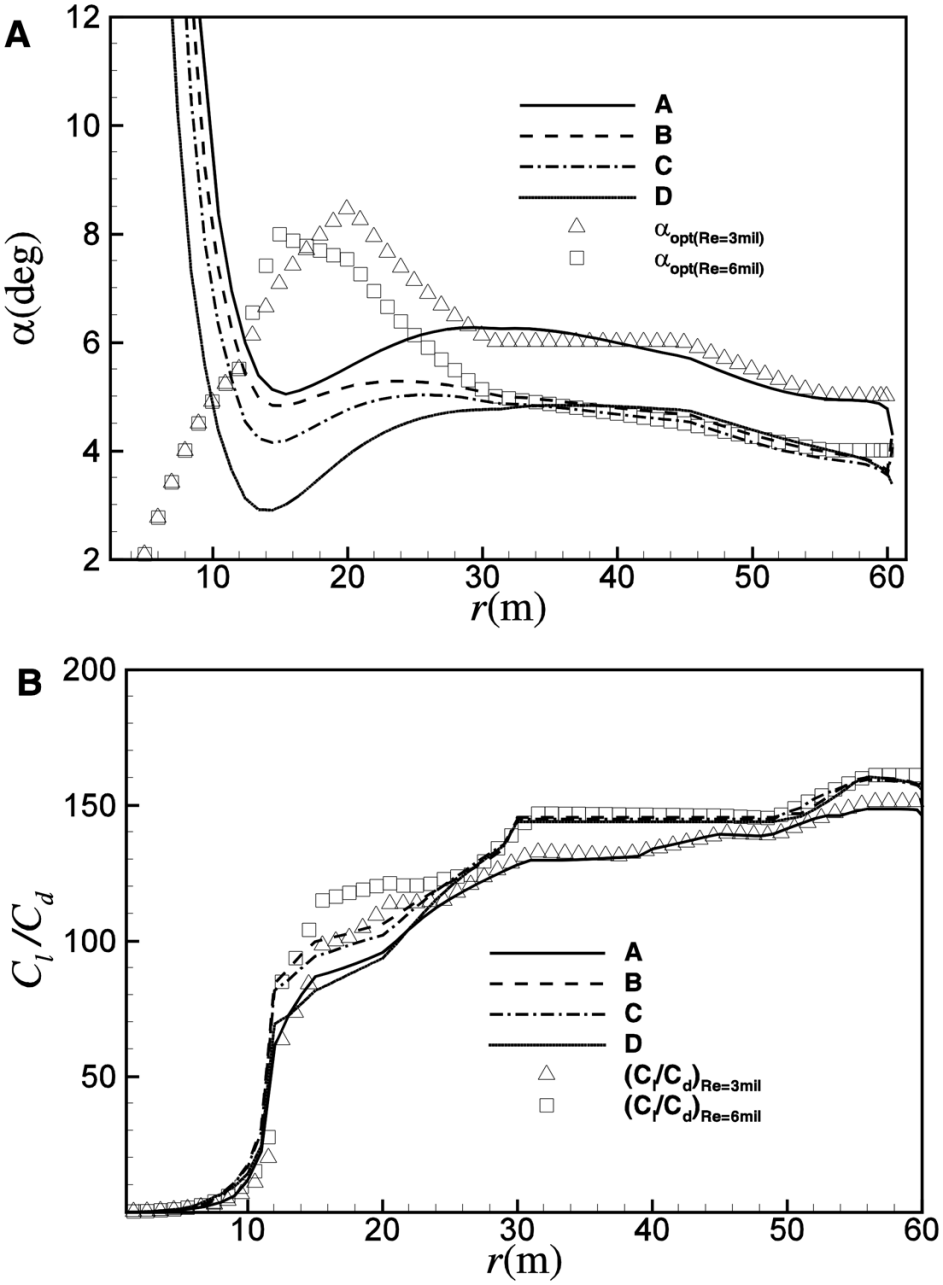
Distributions of (A) *α* and (B) *C*_*l*_/*C*_*d*_ for designs of A and B at *λ*_*opt*_.

**Fig 8:**
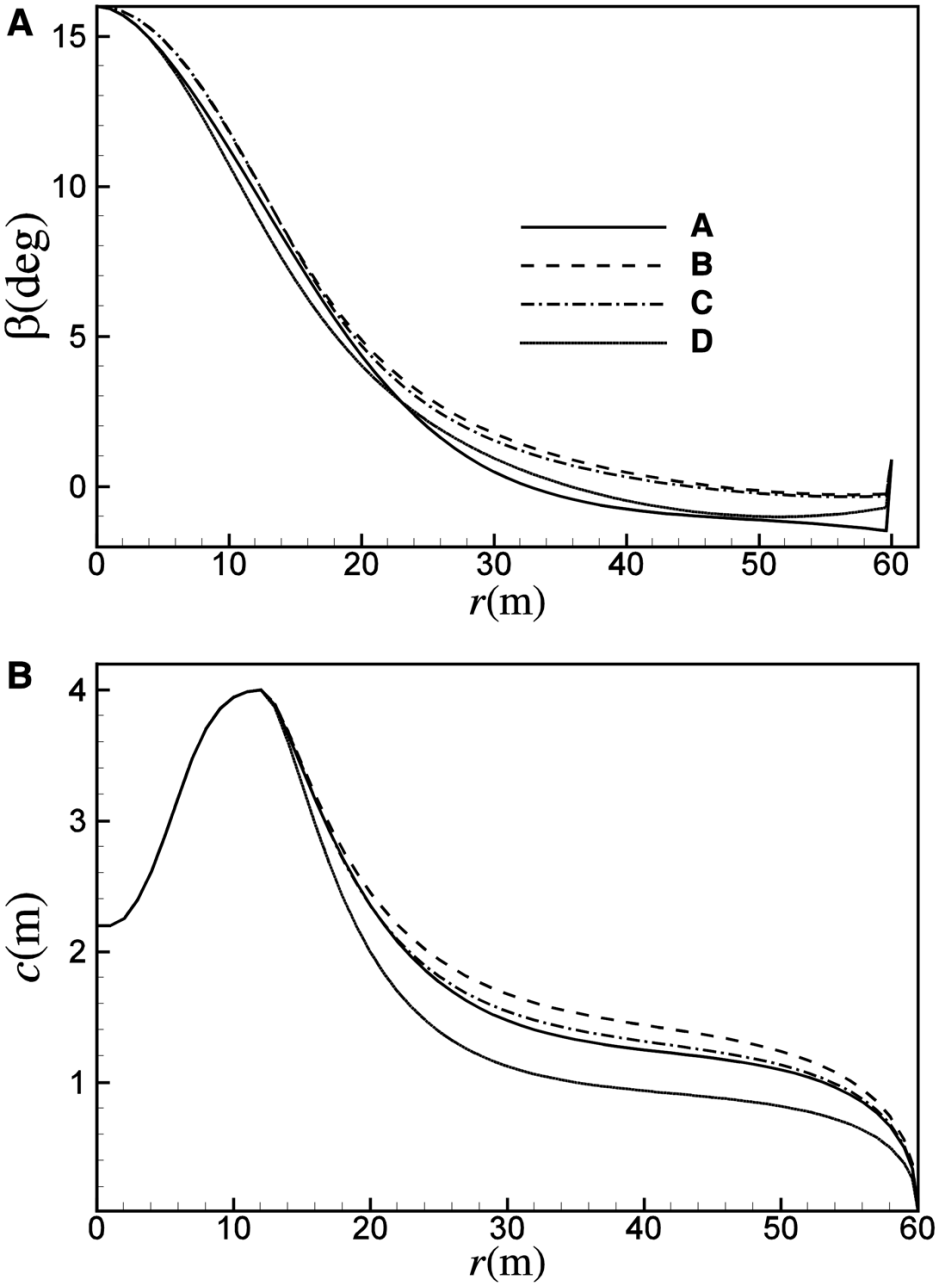
Distributions of (A) twist angle and (B) chord length for design points of A, B, C and D.

Interestingly, although some constraints are implemented artificially in both the chord length and twist angle, the operating *α* and *C*_*l*_/*C*_*d*_ show a good agreement with the ideal condition, as shown in Fig 7. It is observed that in multi-objective optimization of the practical blade, distributions of *α* and *C*_*l*_/*C*_*d*_ for both A and B are well in agreement with the ideal condition at *λ*_*opt*_. As a result, the twist angle of B increases about 1.05 degrees in comparison with that of A, due to that the design *α* is smaller at higher Reynolds number, as shown in Fig 8A. Similarly, due to the increase of the largest *C*_*l*_/*C*_*d*_ at higher Reynolds number, *C*_*Popt*_ increases accordingly. As shown in Fig 6A, *C*_*Popt*_ of A is 0.4918, while *C*_*Popt*_ of B is about 0.497.

Fig 8A shows the distribution of chord length for A and B. Chord length in practical design is mainly dominated by *C*_*l*_ of the thinner airfoils which are arranged post-median of the blade. Compared with A, with the decrease of *C*_*l*_ that corresponding to the best *C*_*l*_/*C*_*d*_ at higher Reynolds number, the chord length for B may increase by up to 9.5%.

**Fig 9:**
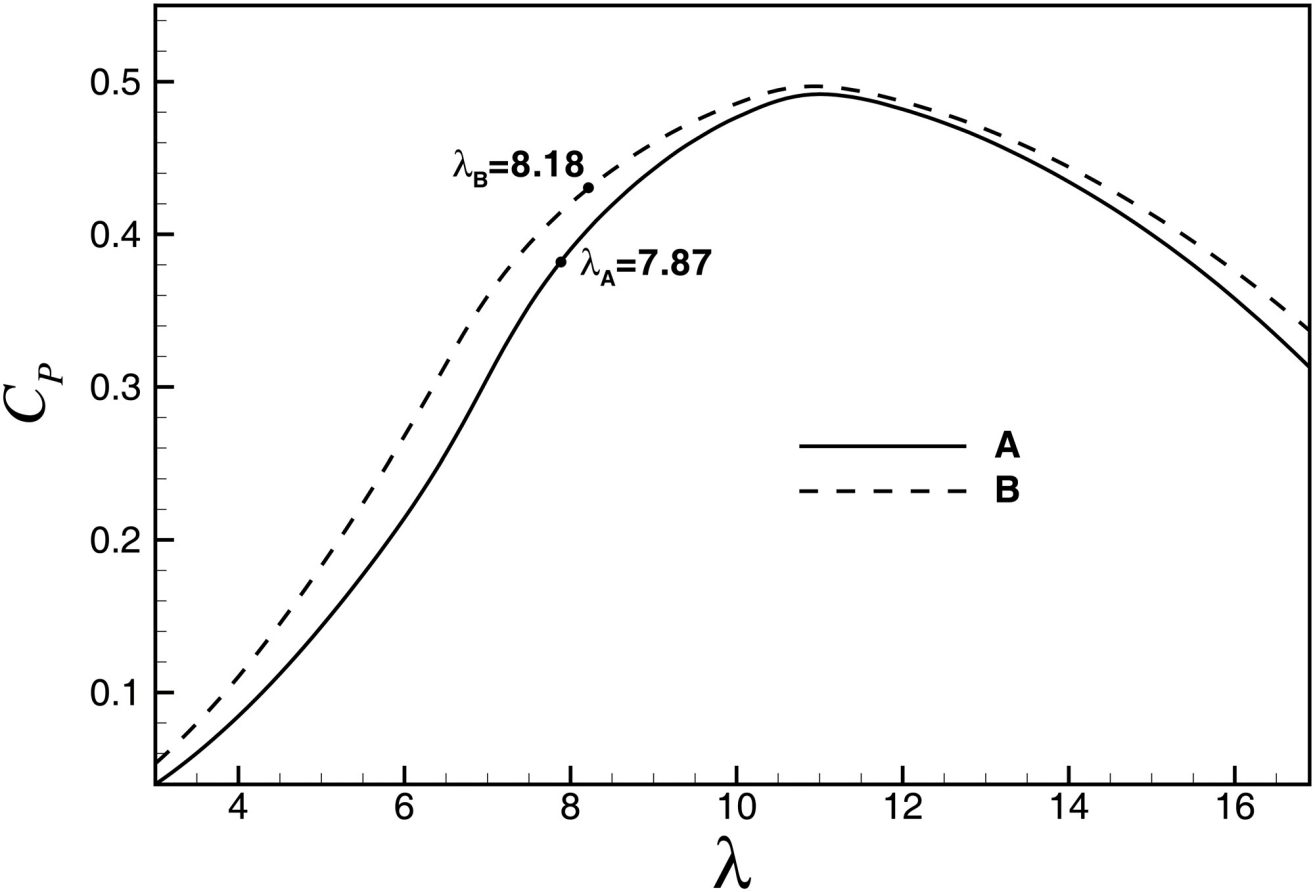
*C*_*P*_-*λ* curves of A and B (pitch angle = 0).

For the variable speed and variable pitch wind turbine, the ultimate *M*_*xy-r*_ generally occurs at the rated wind speed. Fig 9 shows the *C*_*P*_-*λ* curves of A and B. As can be seen, due to the Reynolds number effect, the overall performance of B is better than A. Therefore, compared with A, the rated wind speed of B is slower with a larger *λ* and *C*_*P*_. The rated wind speeds of A and B are 10.3m/s and 9.9m/s when *λ*_*r*_ are 7.87 and 8.18, respectively. For wind turbines with the same configuration, the in-plane load *M*_*x-r*_ is constant, applied by the electric rotor; hence *M*_*xy-r*_ is determined only by out-plane load *F*_*x*_. By applying the momentum theory on each infinitesimal *dr* section of the blade, the distribution of *C*_*P*_ can be written as:

$$C_{P}(r)=\frac{8a{\left(1-a\right)}^{2}rdr}{R^{2}}\text{cos}\theta$$ (18)

Notably, when *λ* <*λ*_*opt*_, there is *a*<1/3. The coefficient of out-plane load can be calculated from Eq (19):

$$C_{Fx}(r)=\frac{8a(1-a)rdr}{R^{2}}\text{cos}\theta$$ (19)

Hence, the out-plane load can be solved by Eq (20):

$$F_{x}(r)=\frac{1}{2}\rho U_{\infty }^{2}\pi R^{2}C_{Fx}$$ (20)

**Fig 10:**
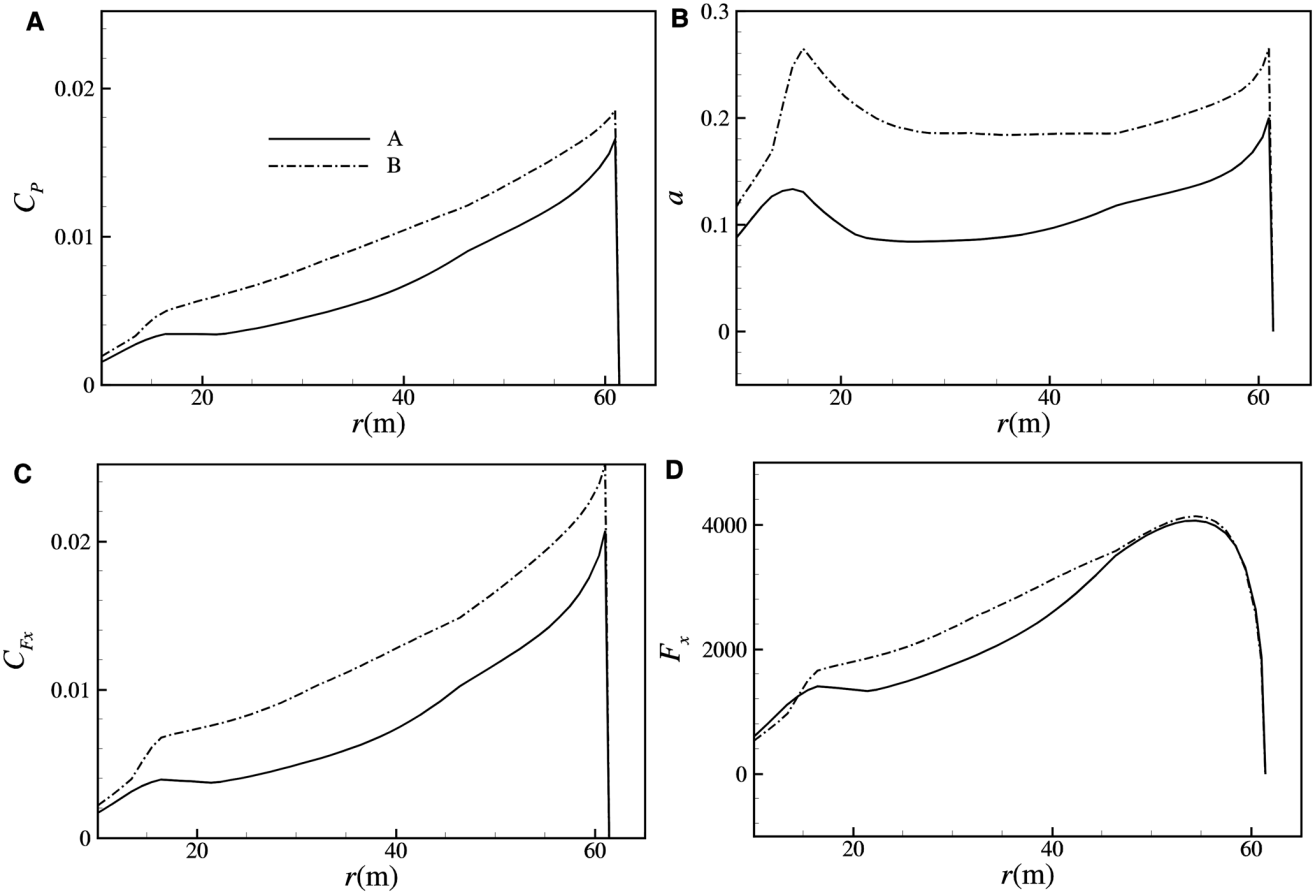
Distributions of (A) *C*_*P*_, (B) axis induction factor *a*, (C) out-plane load coefficient *C*_*Fx*_, (D) out-plane load *F*_*x*_ for designs of A and B at the rated condition.

When *a*⊰(0, 1/3), both *C*_*p*_ and *C*_*Fx*_ monotonically increase with *a*. Therefore, at the rated point, for *C*_*PB*_ >*C*_*PA*_, it can be obtained that *a*_*B*_ *>a*_*A*_, and *C*_*Fx-B*_>*C*_*Fx-A*_, as shown in Fig 10A and 10C. Although *V*_*rB*_<*V*_*rA*_, the ultimate *M*_*xy-r*_ of B is still about 5.2% larger than that of A, due to the huge gap of *C*_*Fx*_ between A and B. It is shown from the above analysis that at the largest *C*_*Popt*_, due to the larger operating *C*_*P*_ at the rated condition, a larger ultimate *M*_*xy-r*_ is induced at higher Reynolds number.

### 3.2 Comparison of A and C/D

As shown in Fig 6A, point C represents the design on Pareto frontier at Re = 6×10^6^ with the same ultimate *M*_*xy-r*_ as A, and D represents that with the same *C*_*Popt*_ as A. Different from B, C and D are designs with larger *λ*_*opt*_. For an ideal blade, the chord length is determined by both the *λ*_*opt*_ and corresponding *C*_*l*_ based on Eq (16). Unlike the variation between A and B, *λ*_*opt*_ of A and C/D are very different. Here, *λ*_*opt*_ of A, C and D are 10.9, 11.5 and 13.4, respectively. Fig 8A gives distributions of the angle of attack of C and D under the optimal conditions, which are well in agreement with the ideal angle of attack. Though the design *α* and *C*_*l*_ of thinner airfoils are smaller, the chord length reduces greatly due to the significant increase of *λ*_*opt*_. In comparison with A, the chord length for C may increase by about 5%, and the chord length for D may reduce by about 27%, as shown in Fig 8B. For C/D, due to the increase of *λ*_*opt*_, the inflow angle of the blade element decreases when compared with B. Subsequently, the twist angle of C/D becomes smaller, so as to keep the optimal angle of attack, as shown in Fig 8A.

**Fig 11:**
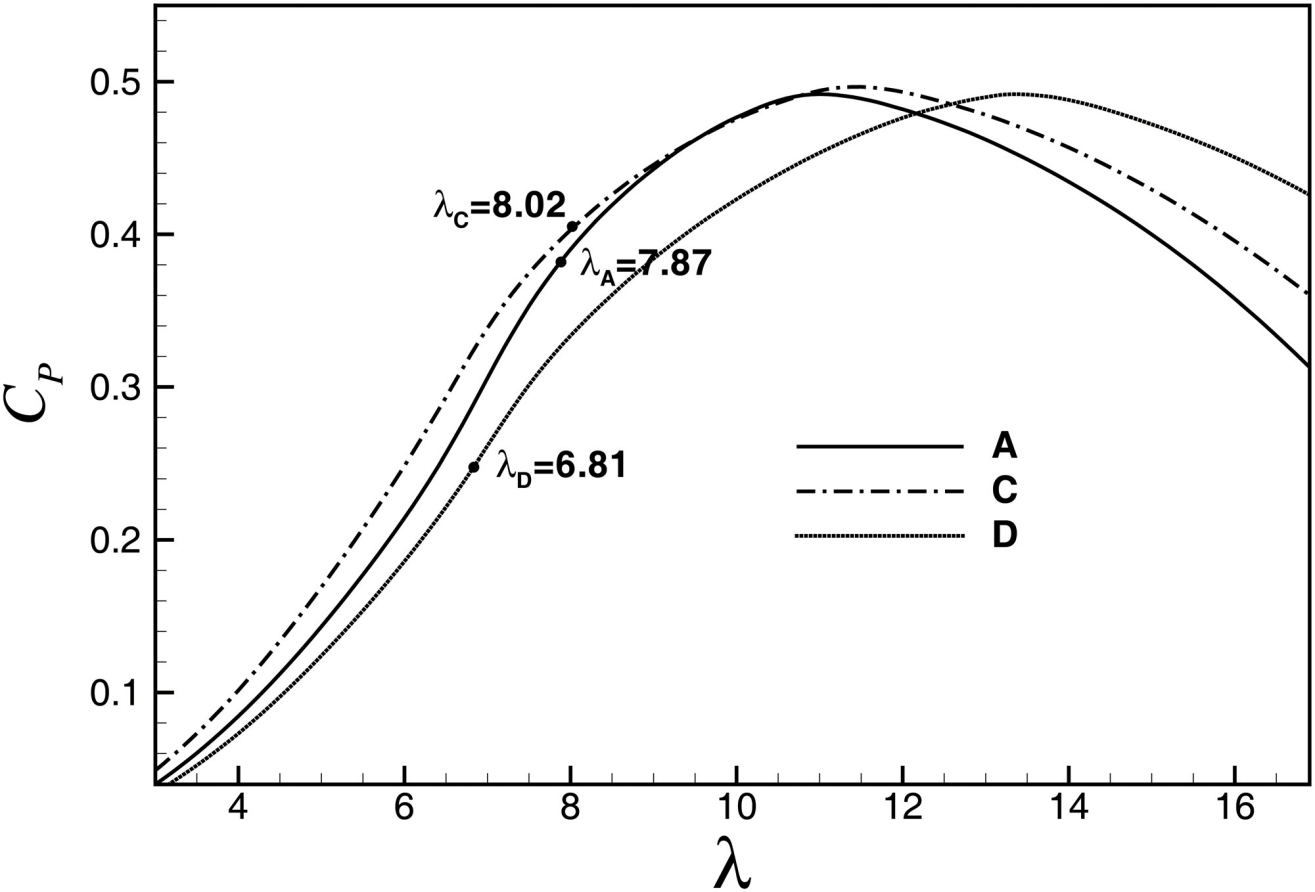
*Cp-λ* curves of A, C and D (pitch angle = 0).

Fig 11 shows the *C*_*P*_-*λ* curves of A, C and D. For designs of A and C, we have *M*_*xy-r*_(A) = *M*_*xy-r*_(C), while for A and D, there is *C*_*Popt*_(A) = *C*_*Popt*_(D) = 0.4918. Since the drag losses increase with *λ*_*opt*_, we have *C*_*Popt*_(C)>*C*_*Popt*_(D). As shown in Fig 6A, compared with designs of A/D, *C*_*Popt*_ for the design of C may increase about 0.9%.

At the rated point, there is *C*_*PC*_>*C*_*PA*_, thus we have *a*_*C*_ *>a*_*A*_, and *C*_*Fx-C*_>*C*_*Fx-A*_. And meanwhile, we also have *V*_*r-A*_<*V*_*r-C*_. As a result, the same load is sustained by the joint action of *C*_*Fx*_ and *V*_*r*_. For A and D, similar to B and A, the ultimate *M*_*xy-r*_ is dominated by *C*_*Fx*_. As can be seen from Fig 6A, the ultimate *M*_*xy-r*_ of D reduces by about 18%, compared with that of A.

### 3.3 Comparison of Pareto frontiers at *Re* = 3×10^6^ and *Re* = 6×10^6^

The analysis shows that the Reynolds number effect is quite significant on the aerodynamic design of a wind turbine blade. In optimization where *C*_*Popt*_ is strongly emphasized to achieve the maximum *C*_*P*_ at a single point, the design points tend to cover the largest *C*_*l*_/*C*_*d*_ of airfoil sections. Hence, due to the change of design *C*_*l*_ and *α*, both the distributions of chord length and twist angle differ greatly at different Reynolds numbers. The change of *C*_*Popt*_ is mainly attributed to the variation of *C*_*l*_/*C*_*d*_ related with Reynolds number. As shown in Fig 6A, on the whole, the Pareto frontier at Re = 6×10^6^ locates slightly above the Pareto frontier at Re = 3×10^6^, only about 0.4%-1.0% in the coordinate of *C*_*Popt*_. However, the gap from right to left between the two Pareto frontiers is quite large, about 3%-18% in the coordinate of *M*_*xy-r*_. It is worth noting that the influence of Reynolds number is quite different for different points on the Pareto frontiers. At the same load, there is a much bigger difference in *C*_*Popt*_ of the design points on two Pareto frontiers in the region of high *C*_*Popt*_ than in the region of low *C*_*Popt*_. Thus, at the same load, *C*_*Popt*_ of E is only about 0.4% smaller than F, while *C*_*Popt*_ of A is about 0.9% smaller than C. Similarly, at the same *C*_*Popt*_, there is a much bigger variation in loads of the design points on two Pareto frontiers in the region of high load than in the region of low load. Thus, at the same *C*_*Popt*_, the ultimate load of D is about 18% smaller than A, while the load of G is only about 3% smaller than E. On the whole, it can be observed that the influence of Reynolds number on Pareto frontier is quite small in the aspect of *C*_*Popt*_. But the characteristics of Pareto frontier leads to a substantial change in load. On the Pareto frontier, *C*_*Popt*_ and *M*_*xy-r*_ are contradictories; any profit of *C*_*Popt*_ is obtained at the cost of a much larger increase in the ultimate load. Take B and C as an example, compared with the design point C, *C*_*Popt*_ increases by 0.1% at the point of B, but *M*_*xy-r*_ increases about 4.4%. Hence, if Pareto frontier at a higher Reynolds number still keeps the same maximum *C*_*Popt*_ as that at a lower Reynolds number, a quite significant load will be saved. Here, 18% is the saved load if a same *C*_*Popt*_ as A is obtained on Pareto frontier at Re = 6×10^6^. And it is also the cost of load, to obtain the profit of *C*_*Popt*_ by 0.9% from D to C. Hence, due to the very gentle slope of Pareto frontiers, the slight downward/upwards shift of Pareto frontiers leads to a big gap between the two Pareto frontiers on the left and right in the plane of *C*_*Popt*_ *-M*_*xy-r*_.

## Optimization of the Wind Turbine Blade Based on *AEP* and *M*_*xy-r*_ at *Re* = 3×10^6^ and *Re* = 6×10^6^

Although *C*_*Popt*_ is an important indicator of wind turbine power efficiency, the cost of energy is our ultimate concern. Hence in this section, *AEP* is set to be one of the two objectives in optimization, instead of *C*_*Popt*_.

### 4.1 Comparison of Pareto frontiers at *Re* = 3×10^6^ and *Re* = 6×10^6^

**Fig 12:**
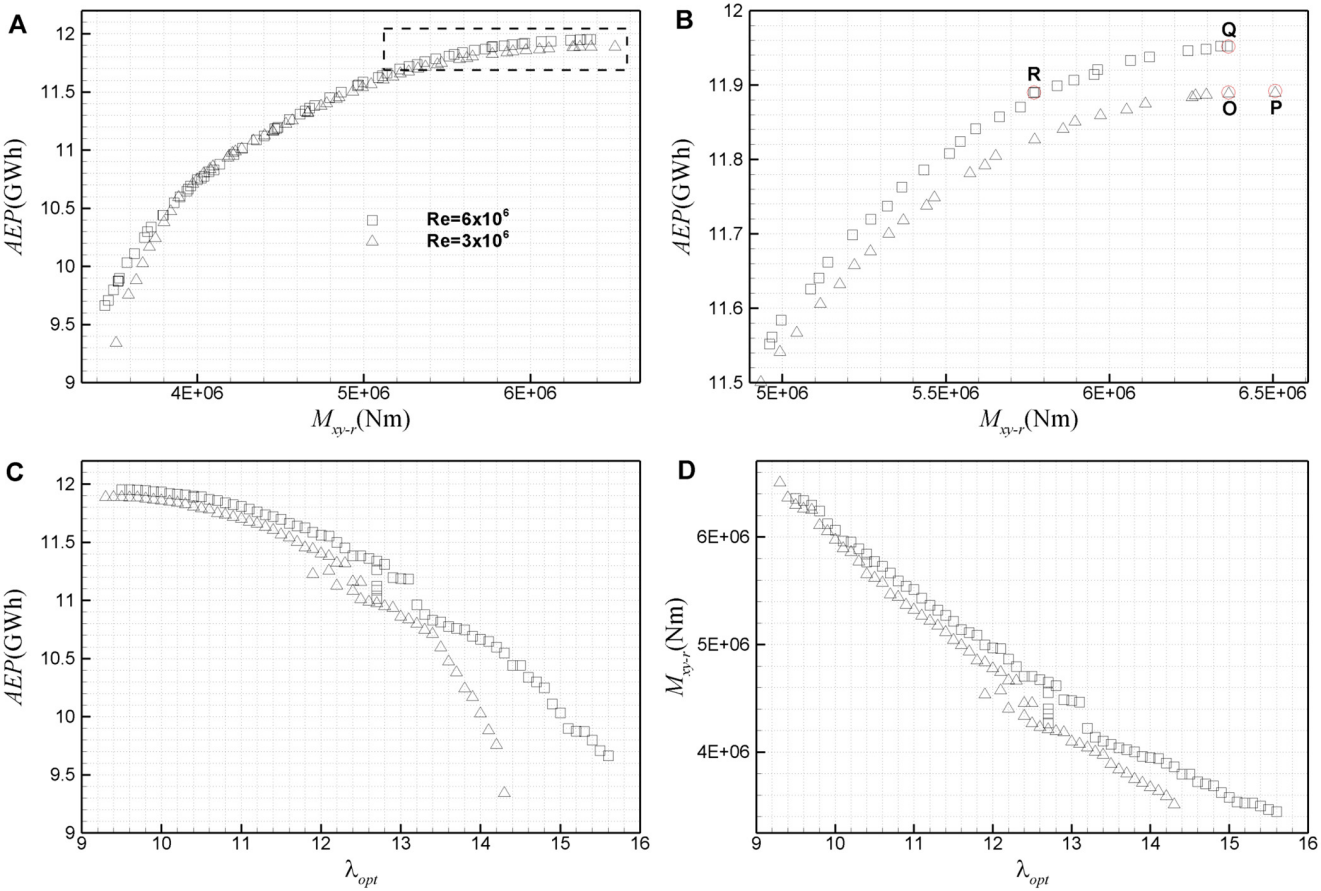
Pareto frontiers of the optimization based on ultimate *M*_*xy-r*_ and *AEP*. (A) total view of Pareto frontier in the plane of *AEP-M*_*xy-r*_, (B) partial enlarged view of Pareto frontier, (C) Pareto frontier in the plane of *AEP*-*λ*_*opt*_, (D) Pareto frontier in the plane of *M*_*xy-r*_ *-λ*_*opt*_.

The results of multi-objective optimization based on *AEP* and *M*_*xy-r*_ are shown in Fig 12. Pareto frontiers exhibit a relative position similar to that in Fig 6A, especially in the region of high *AEP*, which means that the design based on airfoil database at a higher Reynolds number has a larger *AEP* at the same ultimate load *M*_*xy-r*_, or has a smaller load at the same *AEP*. Compared with Fig 6A, the gap between the two Pareto frontiers in Fig 12 is obviously smaller, especially in the region of 4×10^6^<*M*_*xy-r*_<5×10^6^, where the Reynolds number effect is very little. In the present discussion, it is mainly concerned with the right side of Pareto frontiers with *M*_*xy-r*_>5×10^6^, because the design points here are usually selected in practical design due to their high *AEP*. In this region, *AEP* of the Pareto frontier at Re = 6×10^6^ is about 0.2–0.5% larger than that at Re = 3×10^6^. But at the same *AEP*, loads of the two Pareto frontiers vary greatly, about 1%-10%. Similar to Pareto frontiers based on *M*_*xy-r*_ and *C*_*Popt*_, when *AEP* achieves a comparatively large value, any profit of *AEP* must be obtained at the cost of a much larger increase in load. Hence, in the region of high *AEP*, a slight change in *AEP* leads to a significant change in load, due to the change of Reynolds number.

**Fig 13:**
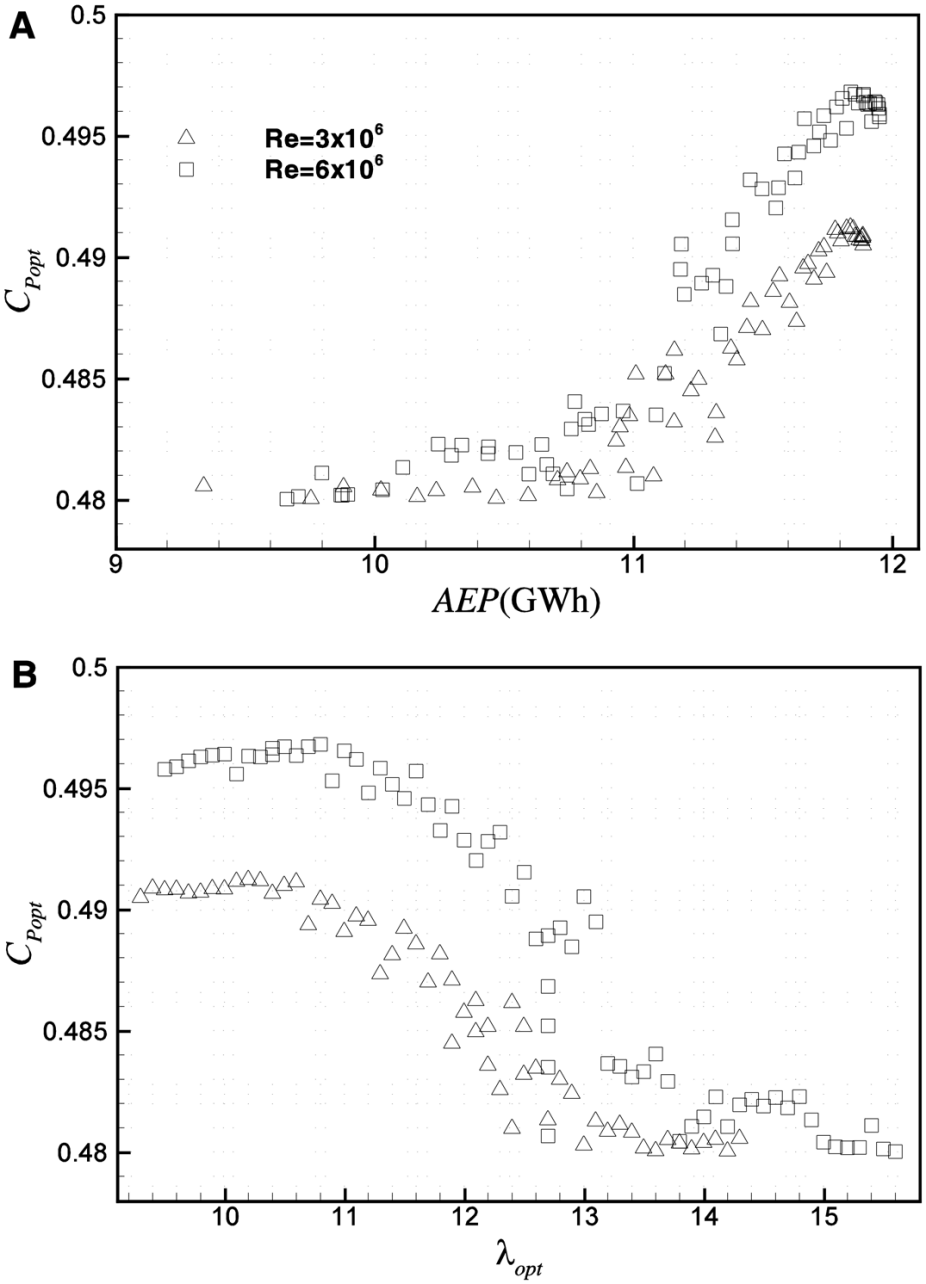
Pareto frontiers based on *M*_*xy-r*_ and *AEP* in planes of (A) *C*_*popt*_ *-AEP*, (B) *C*_*popt*_-*λ*_*opt*_.

Fig 12C and 12D shows the *AEP* and load of Pareto frontiers against *λ*_*opt*_. In general, both the *AEP* and load decrease with *λ*_*opt*_, quite similar to the first kind optimization. But the design points with *λ*_*opt*_<10.9 in this optimization, which have a larger *AEP* and load, are missed in the first kind optimization, which is shown if Fig 6B. Furthermore, Pareto frontiers based on *M*_*xy-r*_ and *AEP* are plotted in both the planes of *C*_*popt*_ *-AEP* and *C*_*popt*_-*λ*_*opt*_ in Fig 13. As can be seen, in optimization, *AEP* and *C*_*popt*_ do not have a positive correlation in a strict sense. Conversely, at a high value of *AEP*, *C*_*popt*_ decreases with *AEP*. Hence, in the optimization based on *C*_*Popt*_ and *M*_*xy-r*_, designs with lower *C*_*Popt*_ and *λ* but larger *AEP* and load are missed. Therefore, in aerodynamic design of a wind turbine blade, special attention should be paid to *λ*_*opt*_ which is slightly lower than the very *λ*_*opt*,_ to achieve the maximum *C*_*Popt*_, because in this region, blades usually have a larger *AEP* but smaller *C*_*Popt*_.

To more clearly reveal the Reynolds effect on multi-objective optimization, several representative design points are selected in Fig 12B for comparison. Points P and Q correspond to designs with the maximum *AEP* on Pareto frontiers at Re = 3×10^6^ and Re = 6×10^6^, respectively. Compared with P, the load of Q is about 2.3% smaller, while *AEP* is about 0.51% larger. Point O corresponds to the design on Pareto frontier at Re = 3×10^6^ with the same load as Q. Point R is the design on Pareto frontier at Re = 6×10^6^ with the same *AEP* as O. Compared with O, *AEP* of Q is about 0.53% larger, and the load of R is about 9.4% smaller.

### 4.2 Comparison of O, P, Q and R

**Fig 14:**
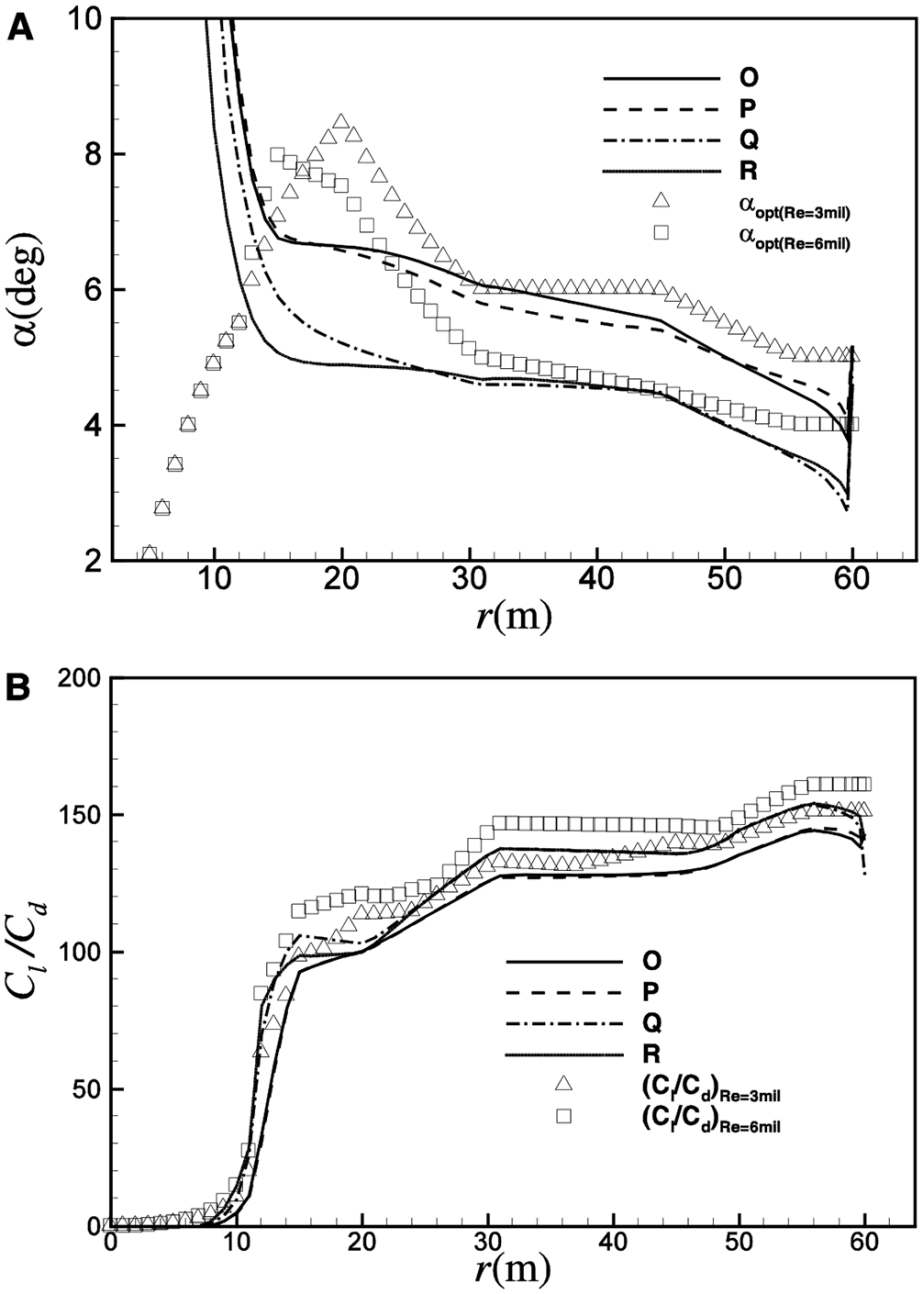
Distributions of (A) *α* and (B) *C*_*l*_/*C*_*d*_ for designs of O, P, Q and R at the corresponding *λ*_*opt*_.

**Fig 15:**
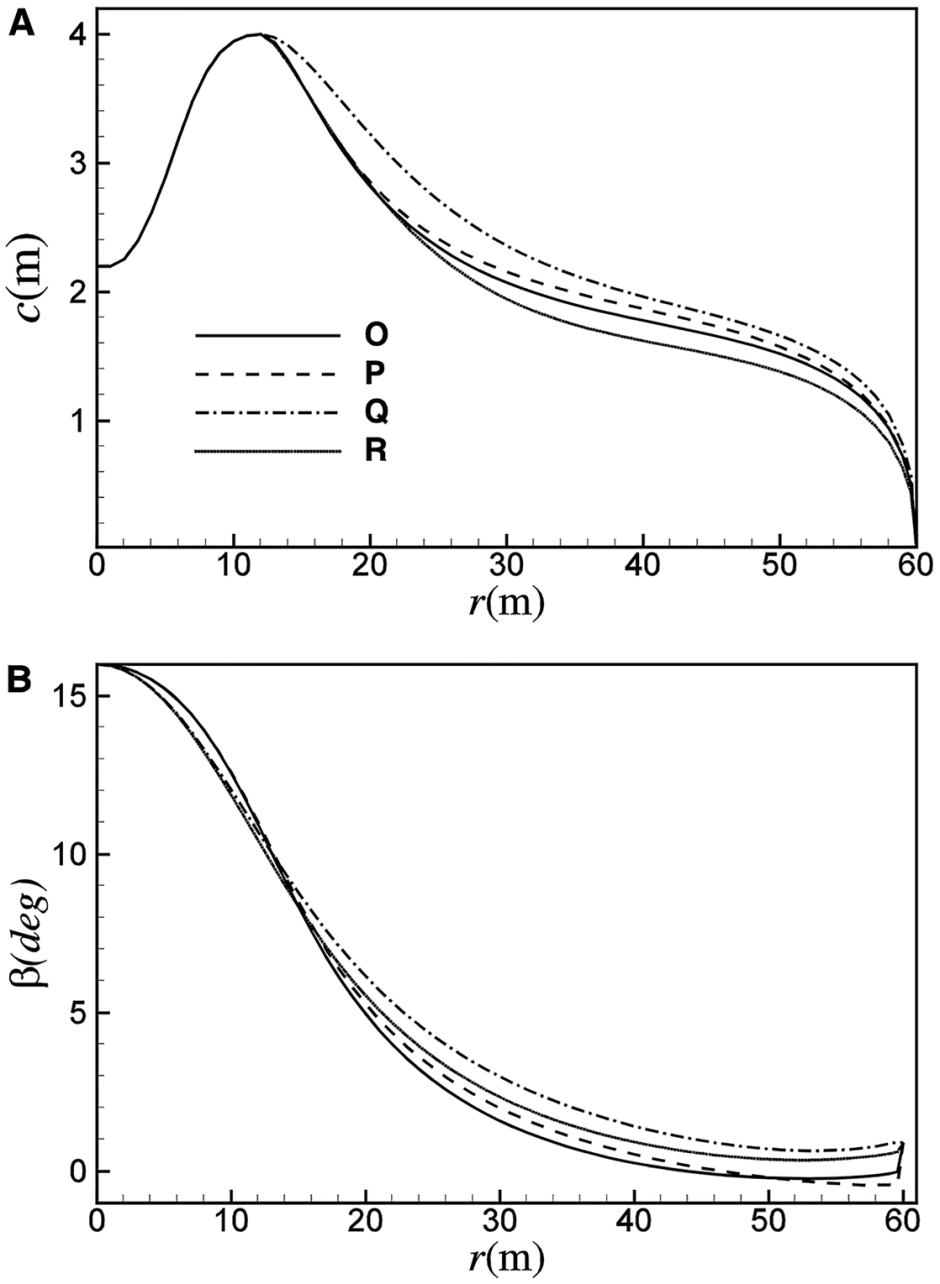
Distributions of (A) chord length and (B) twist angle for design points of O, P, Q and R.

Fig 14 shows the distribution of *α* and *C*_*l*_/*C*_*d*_ for O, P, Q and R at the corresponding *λ*_*opt*_. Different from the optimization based on *C*_*Popt*_, both the operating *α* and *C*_*l*_/*C*_*d*_ show a certain degree of deviation from the optimal conditions. Generally, at *λ*_*opt*_, the blade element operates at an angle of attack that is a little smaller than the optimal conditions, and thereby a smaller *C*_*l*_/*C*_*d*_. Fig 15 shows the distribution of chord length and twist angle for the four design points. A similar trend as Fig 8 can be observed. For the smaller operating *C*_*l*_ of Q, the chord length of Q is about 10% larger than P. In comparison with O, R can achieve a reduction in chord length of about 9%. As shown in Fig 15B, for the larger operating angle of attack at smaller Reynolds number, the twist angle of P is about 1.0 degree smaller than Q, and the twist angle of R is about 0.8 degree smaller than O.

## Design Uncertainty with Mismatched Reynolds Number

The above results indicate that Reynolds number can substantially affect the aerodynamic design of wind turbine rotors. Therefore, think about what will happen if an airfoil database with mismatched Reynolds numbers is used in design? Also, two kinds of mismatched designs should be taken into consideration: the first kind is a wind turbine rotor design using an airfoil database at higher Reynolds numbers than the practical operating condition, while the second kind is a wind turbine rotor design using an airfoil database at lower Reynolds number than the actual one. In this section, both kinds of multi-objective optimization at mismatched Reynolds numbers are to be discussed.

**Fig 16:**
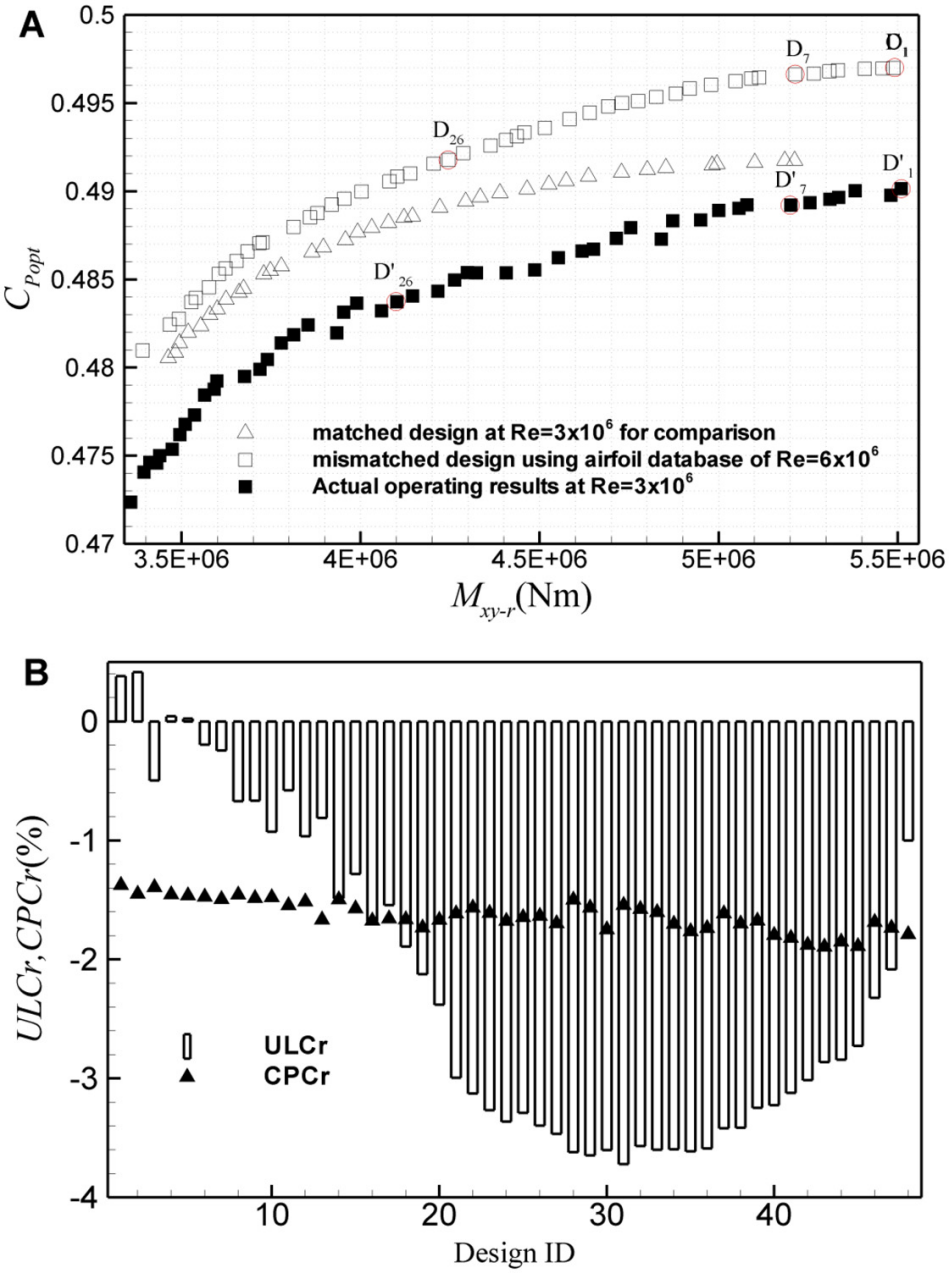
Results of the first kind of mismatched design based on the ultimate *M*_*xy-r*_ and *C*_*Popt*_. (A) Pareto frontiers of the mismatched design and the practical operating results, (B) excursion of the practical operating *M*_*xy-r*_ and *C*_*Popt*_ from the mismatched design values.

**Fig 17:**
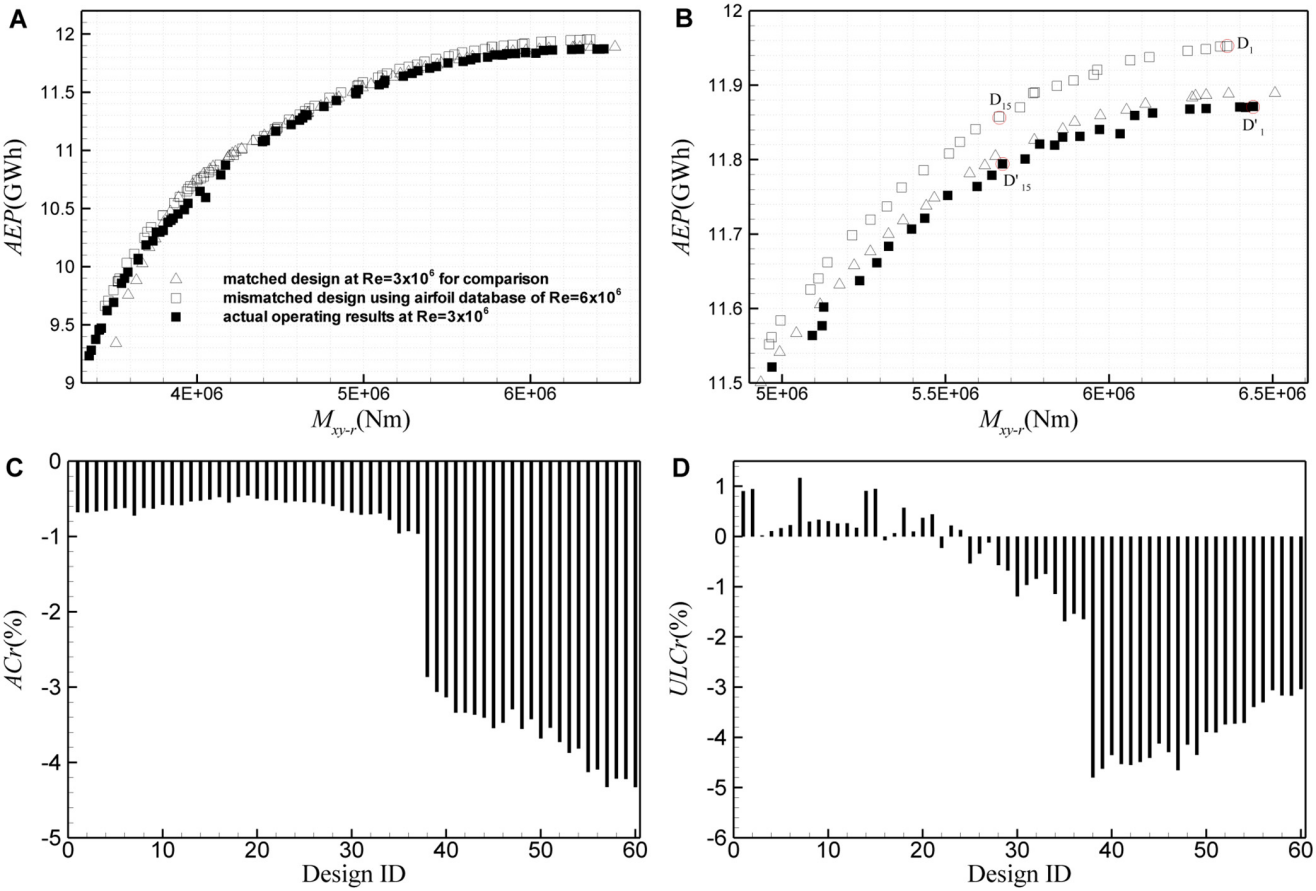
Results of the first kind of mismatched design based on the ultimate *M*_*xy-r*_ and *AEP*. (A) Pareto frontiers of the mismatched design and the practical operating assessment, (B) the partial enlarged view for design ID<30, (C) excursion of the practical *AEP* from the mismatched design value, (D) excursion of the practical ultimate *M*_*xy-r*_ from the mismatched design value.

To study the first kind of mismatched design, it is assumed that a wind turbine rotor practically running at Re = 3×10^6^ is designed using the airfoil database at Re = 6×10^6^. Fig 16 gives the results of this kind of mismatched design based on the ultimate *M*_*xy-r*_ and *C*_*Popt*_. To show clearly, the matched design results and the actual operating assessment of mismatched designs are given for comparison. The three design points D_1_, D_2_, and D_3_ on mismatched Pareto frontier and the corresponding points D’_1_, D’_2_, and D’_3_ of actual operating assessment are also marked out for clarity. Here, the subscript is ID of the design points, and ID is the sequence of design points from right to left on Pareto frontier. It can be seen that both the ultimate *M*_*xy-r*_ and *C*_*Popt*_ of actual operating assessment exhibit an obvious excursion from the mismatched design value. Here, the deviation can be attributed to two factors: one is the Reynolds effect on airfoil performance, which is shown in detail in Section 2.2. Due to the worse performance at lower Reynolds number, even though the blade design is optimized, the operating performance is still worse than the mismatched design value at higher Reynolds number, which can be seen from the gap between the two frontiers at two different Reynolds numbers. The other is the design deviation from the optimal shape. Since the airfoil database does not match the actual operating condition, the distributions of chord length and twist angle deviate from the optimal results. Thus, the expected load and aerodynamic efficiency cannot be obtained, which can be seen from the gap between the matched design and the actual operating assessment. Furthermore, the change rate of the ultimate *M*_*xy-r*_ and *C*_*Popt*_ from the mismatched design values to the operating values based on airfoil data with correct Reynolds number, represented by *ULCr* and *CPCr*, is shown in Fig 16B, respectively. As can be seen, in comparison with the actual operating condition, both the ultimate *M*_*xy-r*_ and *C*_*Popt*_ for most design points are slightly overestimated. *C*_*Popt*_ is overestimated by about 1.5%, while the ultimate *M*_*xy-r*_ is overestimated by about 3.8% in maximum, only except for several design points with small ID, with the loads being underestimated by less than 0.5%. Fig 17 shows the results of the first kind of mismatched design based on the ultimate *M*_*xy-r*_ and *AEP*. Similar to Fig 16, it can also be observed an obvious excursion of the practical ultimate *M*_*xy-r*_ and *AEP* from the mismatched design values. For the design ID<30, the Reynolds number effect is rather little, the estimation error of *M*_*xy-r*_ is less than 1.2%, and *AEP* is overestimated by less than 1%. But for the design ID>35, both the ultimate *M*_*xy-r*_ and *AEP* are significantly overestimated. In this region, *AEP* is overestimated by up to 4.5%, while the ultimate *M*_*xy-r*_ is overestimated by about 5% in maximum. If it happens, the overestimation of *AEP* and load will substantially increase the risk of revenue of a wind energy project, and increase the design cost of a wind turbine for the manufacturer.

**Fig 18:**
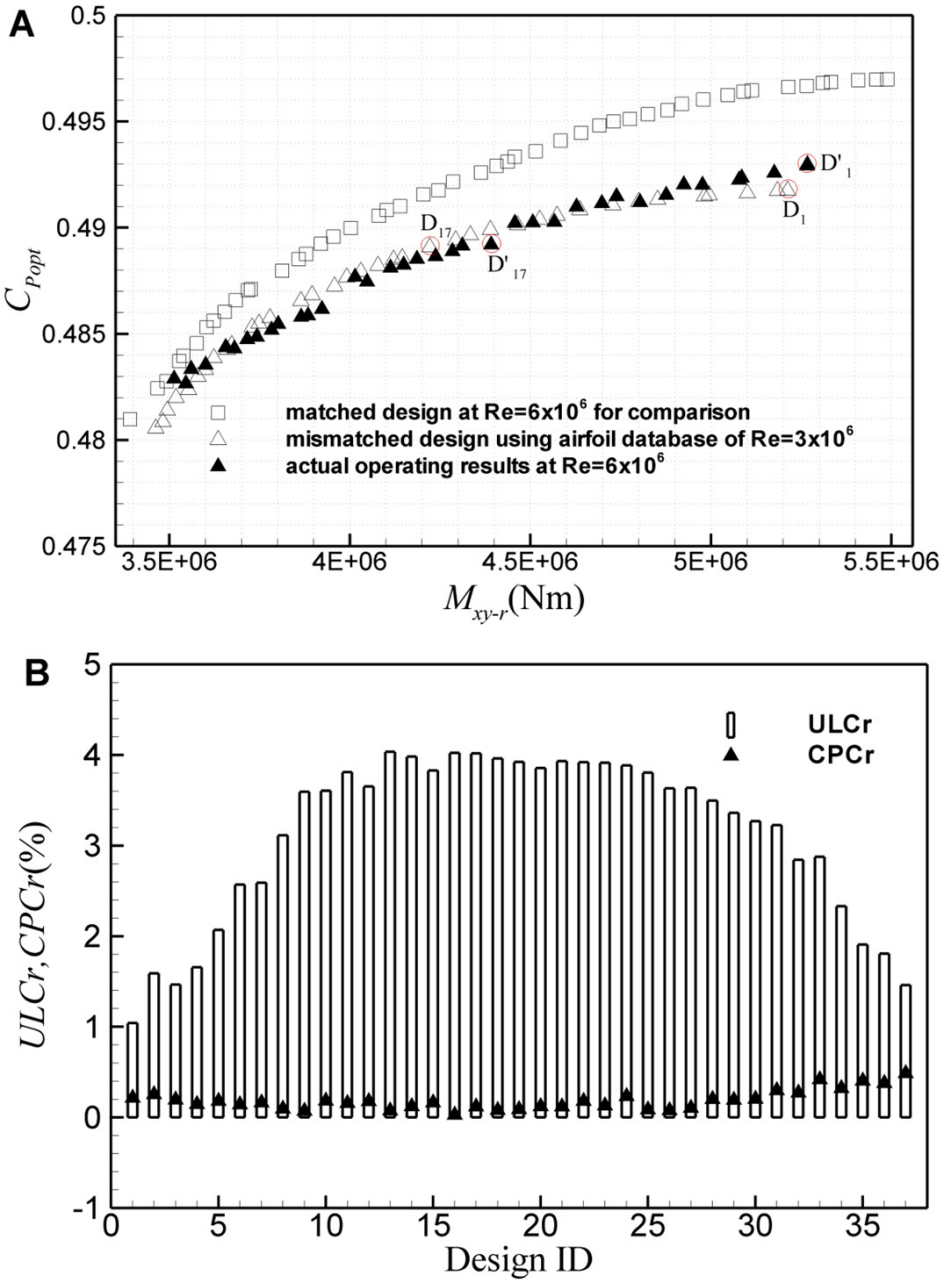
Results of the second kind of mismatched design based on the ultimate *M*_*xy-r*_ and *C*_*Popt*_. (A) Pareto frontiers of the mismatched design and the practical operating assessment, (B) excursion of the practical operating *C*_*Popt*_ and *M*_*xy-r*_ from the mismatched design values

**Fig 19:**
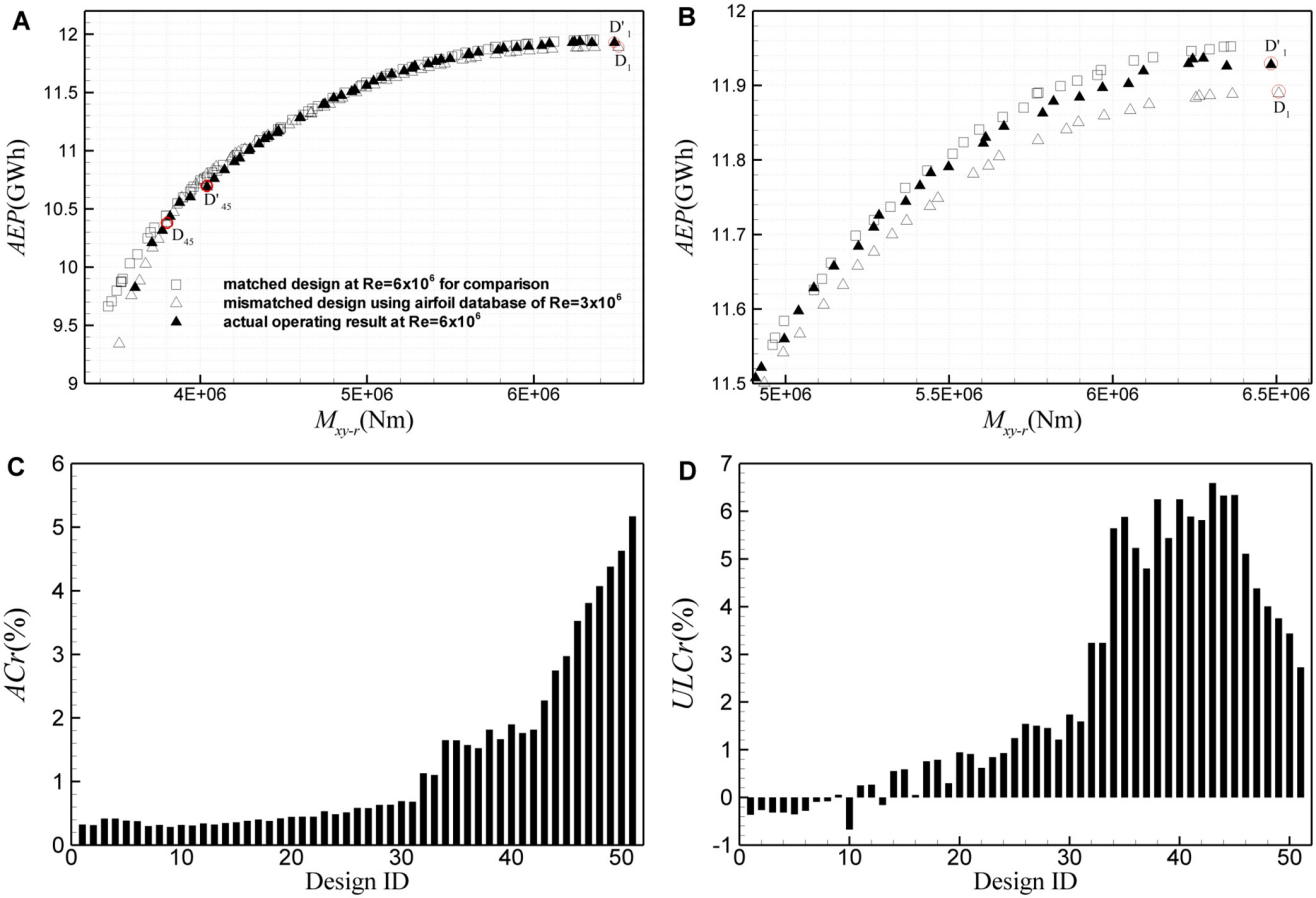
Results of the second kind of mismatched design based on ultimate *M*_*xy-r*_ and *AEP*. (A) Pareto frontiers of the mismatched design and the practical operating assessment, (B) the partial enlarged view for design ID<30, (C) excursion of the practical *AEP* from the mismatched design value, (D) excursion of the practical ultimate *M*_*xy-r*_ from the mismatched design value.

To study the second kind of mismatched design, a wind turbine rotor practically running at Re = 6×10^6^ is designed using the airfoil database at Re = 3×10^6^. Fig 18 shows results of the second mismatched design based on the ultimate *M*_*xy-r*_ and *C*_*Popt*_. Similarly, on one hand, due to the Reynolds number effect on airfoil, the performance of the airfoils with the correct Reynolds number is better than the airfoil database used, while on the other hand, due to the mismatched airfoil database, the design deviates from the optimal shape. As a result, the practical operating assessment results deviate from the design values. However, unlike the first kind of mismatched design, the practical assessment results cannot be enveloped by the design frontier, as shown in Fig 18A. Change rates of the ultimate *M*_*xy-r*_ and *C*_*Popt*_ for each design point are also given in Fig 18B. As is shown, in comparison with the design value, the actual operating *C*_*Popt*_ changes very slightly. However, in some cases, load of the practical operating assessment is significantly larger than the design value. The load is underestimated by about 4% in maximum. Fig 19 shows results of the second kind of mismatched design based on the ultimate *M*_*xy-r*_ and *AEP*. For the design ID<30, the Reynolds number effect is still rather little, the estimation error of *M*_*xy-r*_ is less than 2%, and *AEP* is underestimated by less than 0.5%. But for the design ID>35, *AEP* can be underestimated by up to 5.2%, while the ultimate *M*_*xy-r*_ can be underestimated by about 6.5% in maximum.

The results indicate that in some cases, the influence of Reynolds number is quite small for both kinds of multi-objective optimization; while in other cases, a substantial excursion occurs between the actual operating assessment and the design values. In the first kind of mismatched design, to a certain extent, the overestimation of *C*_*Popt*_ /*AEP* will bring some risks to the earnings of a wind farm, while the overestimation of load will increase the design cost of a wind turbine. For the second kind of mismatched design, extensive attention should be paid to the underestimation of load, especially for the manufacturer who wants to design a very large wind turbine but has no airfoil database at enough high Reynolds number, which may bring some undesired risks to the safety of large wind turbines.

## Summary and Conclusions

Multi-objective design of a 60m blade for 3MW wind turbines is performed at Re = 3×10^6^ and Re = 6×10^6^, respectively, using airfoils with a relative thickness ranging from 40% to 18%. To make the study more general, two kinds of optimization are considered: one is based on the ultimate *M*_*xy-r*_ and *C*_*Popt*_, and the other is based on the ultimate *M*_*xy-r*_ and *AEP*. The NSGAII method is introduced for optimization. The results show that for both kinds of multi-objective optimization, Pareto frontiers at higher Reynolds number envelope those at lower Reynolds number. The design point on Pareto frontier at higher Reynolds number tends to have a larger *C*_*popt*_//*AEP* at the same ultimate load *M*_*xy-r*_, or a smaller load at the same *C*_*popt*_ *//AEP*. At the same *M*_*xy-r*_, the influence of Reynolds number on *C*_*popt*_//*AEP* is rather small, but at the same *C*_*popt*_//*AEP*, the loads differ greatly due to the very gentle slope of Pareto frontiers in the region of high *C*_*popt*_//*AEP*.

For the optimization emphasizing *C*_*popt*_, the blades tend to cover the largest *C*_*l*_/*C*_*d*_ of the airfoil sections. Hence, at different Reynolds numbers, both the distributions of chord length and twist angle differ greatly due to the change of design *C*_*l*_ and *α*. For the optimization aiming for *AEP*, the design points with smaller *C*_*Popt*_ and *λ*_*opt*_ but larger *AEP* and load are captured, compared with the former optimization. In the latter optimization, the blade elements tend to run at a small *C*_*l*_/*C*_*d*_ for the maximum *AEP*. At an equivalent tip-speed ratio or load, the blade operating at higher Reynolds number tends to have a larger chord length and twist angle for the maximum *C*_*popt*_ or *AEP*.

If a wind turbine blade is designed using an airfoil database with mismatched Reynolds numbers, both the ultimate *M*_*xy-r*_ and *C*_*Popt*_//*AEP* of actual operating assessment will exhibit an excursion from the design values. In some cases, the influence of Reynolds number is rather small. But in other cases, the effect is quite significant. For one extreme case in the present study, *AEP* can be overestimated by 4.5%; while for another extreme case, the load can be underestimated by about 6.5%, which all will bring some unexpected risks to the wind power project.

## Abbreviations

*a*: axial induction factor (dimensionless)
*ACr*: the change rate of *AEP* (%)
*Ar*: aspect ratio (dimensionless)
*AEP*: annual energy production (GWh)
*b*: tangential induction factor (dimensionless)
*c*: chord length of the airfoil (m)
*c_max_*: the maximum chord length of blade (m)
*c_tip_*: the chord length of blade tip (m)
*CPCr*: the change rate of *C*_*Popt*_ (%)
*C_l_*: lift coefficient (dimensionless)
*C_d_*: drag coefficient (dimensionless)
*C_P_*: power coefficient (dimensionless)
*C_Popt_*: optimal power coefficient (dimensionless)
*C_Fx_*: coefficient of *F*_*x*_ (dimensionless)
*C_x_*: normal force coefficient (dimensionless)
*C_y_*: tangential force coefficient (dimensionless)
*F*: tip and root losses factor (dimensionless)
*F_x_*: the out-plane load of blade (N)
*M_x-r_*: the rotational moment of blade root (Nm)
*M_xy-r_*: the moment of blade root (Nm)
*N*: the number of blades
*P*: power (W)
*r*: local radius (m)
*r_cmax_*: local radius of the maximum chord length (m)
*r_tip_*: local radius of the blade tip (m)
*r_tmax_*: local radius of the maximum twist angle (m)
*R*: radius of rotor (m)
Re: Reynolds number (dimensionless)
*ULCr*: the change rate of ultimate load (%)
*U*_∞_: wind speed in the far field (m/s)
*V_r_*: rated wind speed (m/s)
*x_ci_*: horizontal coordinate value of the *i-th* control points for the chord (m)
*y_ci_*: vertical coordinate value of the *i-th* control points for the chord (m)
*x_ti_*: horizontal coordinate value of the *i-th* control points for the twist angle (m)
*y_ti_*: vertical coordinate value of the *i-th* control points for the twist angle (deg)
*β*: twist angle (deg)
*θ*: cone angle of a wind turbine rotor (deg)
*ϕ*: inflow angle (deg)
*α*: angle of attack (deg)
*λ*: tip-speed ratio (dimensionless)
*λ_opt_*: optimal tip-speed ratio (dimensionless)
*λ_r_*: tip-speed ratio at the rated condition (dimensionless)
*μ*: *r*/*R* (dimensionless)
*ν*: kinematic viscosity (kg.m^-1^.s^-1^)
Ω: the rotational speed of rotor (rad/s)
*ρ*: air density (kg/m^3^)
*β_max_*: the maximum twist angle of blade (deg)
